# Implied Maximum Dose Analysis of Standard Values of 25 Pesticides Based on Major Human Exposure Pathways

**DOI:** 10.3934/publichealth.2017.4.383

**Published:** 2017-07-24

**Authors:** Zijian Li, Aaron A. Jennings

**Affiliations:** 1 Department of Civil Engineering, Case Western Reserve University, Cleveland, OH 44106, USA; 2 Present Address: Parsons Corporation, Chicago, IL 60606, USA

**Keywords:** pesticide standard, implied maximum dose limits, human health risk, acceptable daily intake, human exposure

## Abstract

Worldwide jurisdictions are making efforts to regulate pesticide standard values in residential soil, drinking water, air, and agricultural commodity to lower the risk of pesticide impacts on human health. Because human may exposure to pesticides from many ways, such as ingestion, inhalation, and dermal contact, it is important to examine pesticide standards by considering all major exposure pathways. Analysis of implied maximum dose limits for commonly historical and current used pesticides was adopted in this study to examine whether worldwide pesticide standard values are enough to prevent human health impact or not. Studies show that only U.S. has regulated pesticides standard in the air. Only 4% of the total number of implied maximum dose limits is based on three major exposures. For Chlorpyrifos, at least 77.5% of the total implied maximum dose limits are above the acceptable daily intake. It also shows that most jurisdictions haven't provided pesticide standards in all major exposures yet, and some of the standards are not good enough to protect human health.

## 1. Introduction

The European Commission [1] defines a pesticide as something that prevents, destroys, or controls a harmful organism (“pest”) or disease, or protects plants or plant products during production, storage, and transport. The U.S. Environmental Protection Agency (USEPA) [2] defines a pesticide as a matter or mixture of matters applied for preventing, destroying, repelling, or mitigating any pest. Pests can be bacteria, microorganisms, plants, and any other species that are harmful to crops, human beings, and living animals. Pesticides are largely applied worldwide to control pests and they can be classified by function (Table 1).

**Table 1.** Pesticides Classified by Function.

*Pesticide Type*	*Function*	*Example*
Algicides	Kill algae	Copper sulphate
Antifouling agents	Kill or repel organisms that attach to underwater surfaces	SEA-NINE CR2
Antimicrobials	Kill microorganisms (such as bacteria and viruses)	Sulphonamides
Attractants	Attract pests (to lure an insect or rodent to a trap)	Heptyl Butyrate
Biopesticides	Derived from natural materials	Canola oil
Biocides	Kill microorganisms	Trichlor
Disinfectants and sanitizers	Kill or inactivate disease-producing microorganismss	Alcohol
Fungicides	Kill fungi	Mancozeb
Fumigants	Produce gas or vapor intended to destroy pests in buildings or soil	Methyl bromide
Herbicides	Kill weeds	2,4-D
Insecticides	Kill insects and other arthropods	DDT
Miticides	Kill mites	Permethrin
Molluscicides	Kill snails and slugs	Methiocarb
Nematicides	Kill nematodes	Aldicarb
Ovicides	Kill eggs of insects and mites	Hexythiazox
Pheromones	Disrupt the mating behavior of insects	Androstenone
Repellents	Repel pests and birds	Neem oil
Rodenticides	Control mice and other rodents	Warfarin
Defoliants	Cause leaves or other foliage to drop from a plant	Drexel
Desiccants	Kill leaves	Paraquat
Insect growth regulators	Disrupt the molting, maturity from pupal stage to adult, or other life processes of insects	Triflumuron
Plant growth regulators	Inhibit growth and other plant responses	Ethephon

Pesticides are largely used in agricultural, commercial, industrial, home, and garden applications. After applied to the environment, pesticides can be transported to four major environmental sinks which include soil, water, air, and biomass. Pesticides could be absorbed by soil partials and rushed away into river, groundwater, and lake by rain water. Some volatile and semi-volatile pesticides can evaporate into the air and disperse through winds. Moreover, pesticides can bio-accumulate and bio-magnitude into crops, plants, animals, and human beings through food chain [3].

Pesticides are very common in the environment. Human exposure to pesticides can occur through ingestion, inhalation, and dermal contact [4]. The exposure pathways include ingestion of pesticide contaminated food, drinking water, and soil, inhalation of air and soil dust contaminated by pesticides, and dermal contact by swimming and showering in pesticide contaminated water, touching soil and food contaminated by pesticides. Also, infants can exposure to pesticides through ingestion of pesticide contaminated breast milk [5].

Because most pesticides are toxic chemicals, worldwide jurisdictions are taking actions to help manage human health risks caused by pesticides. The actions include regulation of pesticide standard values (PSVs) such as pesticide soil regulatory guidance values (RGVs), pesticide drinking water and air maximum concentration levels (MCLs), and pesticide food maximum residue limits (MRLs). Most jurisdictions regulated PSVs to specify their maximum allowable concentrations in each exposure pathway. PSVs should be regulated and derived based on human health risk model and applied essential toxicological data like acceptable daily intake (ADI) which is the maximum amount of pesticide that can enter human body without adverse health effects. Previous researches have made contributions on regulating worldwide contamination chemical standards. Proctor et al. [6] conducted research on Chromium standard values regarding human health. Davis et al. [7] analyzed Arsenic soil standards. Also, other studies analyzed pesticide standards in soil and drinking water [8]–[13]. Since human exposure to pesticides may occur by many different exposure pathways, it is necessary to examine PSVs in a more comprehensive approach and consider all the major exposures. Therefore, the objective of this study is to evaluate whether PSVs could protect human health based on all major exposures.

## 2. Materials

### 2.1. Worldwide Jurisdictions and PSVs

The materials needed for this research are worldwide jurisdictions and their PSVs, which include pesticide soil RGVs, pesticide drinking water MCLs, pesticides air MCLs, and pesticide agricultural commodity MRLs. These jurisdictions and PSVs were mainly obtained from online data base. Most governments and environmental departments provided the documents on their official websites. Some materials are collected from other sources such as publication journals, environmental conferences, or news reports. Pesticides from worldwide jurisdictions were identified by Chemical Abstracts Service Registry Numbers (CAS No.). A total of 19,421 soil pesticide RGVs from 174 worldwide soil jurisdictions in 50 nations were identified. Also, a total of 5,474 drinking water pesticide MCLs from 145 worldwide jurisdictions in 95 nations were identified. There are at least 90 worldwide jurisdictions provided agricultural commodity pesticide MRLs. Because only the U.S. regulated pesticide air MCLs, the analysis of air PSVs is omitted. These PSVs references and sources were provided in Supplementary Materials.

### 2.2. The Most Commonly Used Pesticides

Based on current and historical usage, a total of 25 pesticides have been selected for IMDL analysis (Table 2). Among these 25 selected pesticides, a total of 11 are Stockholm Convention Persistent Organic Pollutants (POPs) pesticides which were largely applied historically [14]–[16] and the rest are widely used nowadays [17].

**Table 2.** 25 Selected Pesticides Based on Current and Historical Use.

*Pesticide*	*CAS No.*	*Reason for selection*
Aldrin	309-00-2	The Stockholm Convention POP
Chlordane	57-74-9	The Stockholm Convention POP
DDT	50-29-3	The Stockholm Convention POP
Dieldrin	60-57-1	The Stockholm Convention POP
Endrin	72-20-8	The Stockholm Convention POP
Heptachlor	76-44-8	The Stockholm Convention POP
Toxaphene	8001-35-2	The Stockholm Convention POP
Lindane	58-89-9	The Stockholm Convention POP
Endosulfan	115-29-7	The Stockholm Convention POP
Pentachlorophenol	87-86-5	The Stockholm Convention POP
Bromomethane	74-83-9	The Stockholm Convention POP
Glyphosate	1071-83-6	Current high quantity use
Mancozeb	8018-01-7	Current high quantity use
Chlorothalonil	1897-45-6	Current high quantity use
2,4-D	94-75-7	Current high quantity use
Chlorpyriphos	2921-88-2	Current high quantity use
Atrazine	1912-24-9	Current high quantity use
MCPA	94-74-6	Current high quantity use
Dicamba	1918-00-9	Current high quantity use
Metolachlor	51218-45-2	Current high quantity use
Aldicarb	116-06-3	Current high quantity use
Malathion	121-75-5	Current high quantity use
Diazinon	333-41-5	Current high quantity use
Trifluralin	1582-09-8	Current high quantity use
Diuron	330-54-1	Current high quantity use

## 3. Methods

### 3.1. Implied Maximum Dose Limit

IMDL was introduced in this research to examine the pesticide maximum exposure mass loading based on national jurisdictions PSVs from all major exposure pathways. Pesticide implied dose limits (IDLs) were calculated for each exposure pathway as the following, and because only U.S. regulated pesticide air MCLs, the IDL_air_ calculation was omitted.

For drinking water:

$$IDL_{dw}=\left(\frac{EF}{HW}\right)(MCL)(V)$$ (1)

For residential soil:

$$IDL_{soll}=\left(\frac{EF}{HW}\right)\left[\left(\text{RGV}\right)\left(\text{CF}\right)\left(\text{IR}\right)+\left(\text{RGV}\right)\left(\text{CF}\right)\left(ABS_{d}\right)\left(GIABS\right)\right]$$ (2)

For agricultural commodities:

$$IDL_{food}=\left(\frac{EF}{HW}\right)\overset{n}{\underset{i=1}{\sum }}(MRL_{i})(IR_{i})$$ (3)

All IDLs are based on the following set of exposure scenario coefficient values.

EF – Exposure Factor (1) [18];

HW – Human Weight (70 kg) [18];

V – Volume of water intake rate (2 L/day) [18];

CF – Convert Factor (10^6^ mg/kg);

IR – Intake Rate for soil [19];

ABS_d_ – Absorption Factor [19];

GIABS – GastroIntestinal Absorption Factor [19];

IR_i_ – Intake Rate for food i (kg/day) [20].

And IMDL was derived by adding up IDLs from these possible exposures. If a nation regulated more than one PSVs in one of the major exposures, different IMDLs were calculated by combining different IDL with others.

$$IMDL=\left(\frac{EF}{HW}\right)[\left(\text{MCL}\right)\left(\text{V}\right)+\left(\text{RGV}\right)\left(\text{CF}\right)\left(\text{IR}\right)+(\text{RGV})(\text{CF})(ABS_{d})(GIABS)+\overset{n}{\underset{i=1}{\sum }}(MRL_{i})(IR_{i})]$$ (4)

### 3.2. Cumulative Distribution Function (CDF) Analysis

The arithmetic mean (μ), median (m), standard deviation (σ_L_), and geometric mean (μ_G_) were computed for those selected pesticides IMDLs. CDF analysis was applied to illustrate the distribution of IMDLs. IMDL empirical cumulative distribution for each pesticide was shown as follows.

$$\text{P}\left(IMDL_{r}\leq IMDL_{i}\right)\approx \frac{n_{i}}{N};\;\forall i=1,\ldots ,N$$ (5)

IMDL_r_ – a random IMDL;

IMDL_i_ – a known IMDL;

n_i_ – integer rank of IMDL in N known values.

### 3.3. Pearson (r) Correlation Coefficient

Pearson (r) correlation coefficient was calculated in Equation 5 for each selected pesticide IMDL to measure the degree that an IMDL empirical cumulative distribution fits a theoretical lognormal cumulative distribution calibrated with the computed mean and standard deviation statistics.

$$\text{r}=\frac{N\left[\sum E\left({\text{IMDL}}_{i}\right)\times F\left({\text{IMDL}}_{i}\right)\right]-\left[\sum E\left({\text{IMDL}}_{i}\right)\right]\left[\sum F\left({\text{IMDL}}_{i}\right)\right]}{\sqrt{\left[N\sum E{\left({\text{IMDL}}_{i}\right)}^{2}-{\left(\sum E\left({\text{IMDL}}_{i}\right)\right)}^{2}\right]}\left[N{\sum F\left({\text{IMDL}}_{i}\right)}^{2}-{\left(\sum F\left({\text{IMDL}}_{i}\right)\right)}^{2}\right]}$$ (6)

E (IMDL_i_) – probability calculated from IMDL empirical cumulative distribution;

F (IMDL_i_) – probability calculated from IMDL theoretical lognormal cumulative distribution.

### 3.4. IMDL Cluster

CDF analysis was also applied to find IMDL clusters. IMDL cluster is defined as IMDL interval (IMDL_i_ − IMDL_i+M_) with M non-random values. Binomial probability function expressed in Equation 7 was used to compute the randomly occurring cluster probability (Pc).

$$\begin{array}{l} P_{c}[\text{M }IMDL_{s\varepsilon }\left(IMDL_{i},IMDL_{i+M}\right]\\ =\left[\frac{N!}{M!\left(N-M\right)!}\right]{\left[F\left(IMDL_{i+M}\right)-F\left(IMDL_{i}\right)\right]}^{M}\\ {\left\{1-\left[F\left(IMDL_{i+M}\right)-F\left(IMDL_{i}\right)\right]\right\}}^{N-M} \end{array}$$ (7)

## 4. Results

The IMDLs for 25 selected pesticides were analyzed by CDF and compared with the acceptable daily intake (ADI) value which measures the maximum amount of pesticide which can get into the human body without occurring adverse health effects. IMDLs for three pesticides 2,4-D, Chlorpyrifos, and Diazinon were discussed in this study.

### 4.1. 2,4-D IMDL Analysis

**Figure 1.**
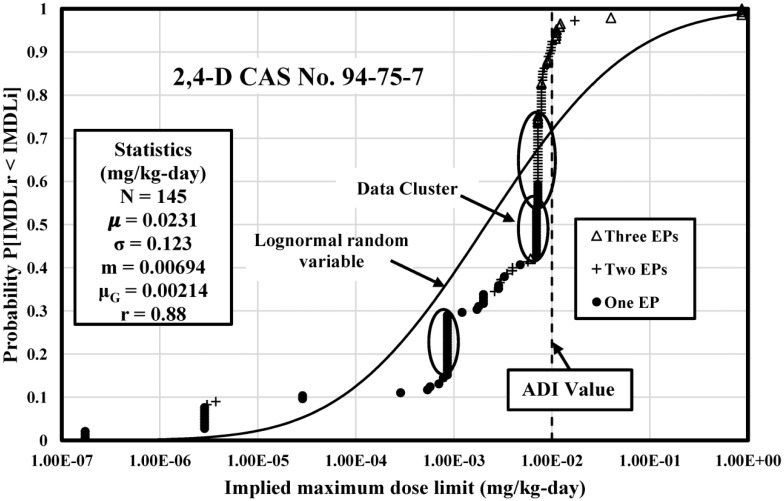
Empirical CDF of 2,4-D IMDLs Computed from Soil, Water, and Agricultural Commodity PSVs Compared to a Theoretical Lognormal CDF with Identical Statistics.

Figure 1 illustrates the CDF of 145 IMDLs calculated from 2,4-D major exposures and compared with a theoretical lognormal CDF calibrated with the computed mean and standard deviation statistics. The IMDLs of 2,4-D range from 1.73 E-07 (Moldova) to 8.66 E-01 mg/kg-day (Vietnam) with 6.70 orders of magnitude. The Pearson correlation coefficient is 0.881, indicating that values are well dispersed over this span. The CDF is skewed by three IMDLs clusters.

The cluster at 7.18 E-03 – 7.81 E-03 mg/kg-day is made up of 33 IMDLs computed from Cambodia, China, Costa Rica, Cuba, Dominican Republic, Honduras, Egypt, Guatemala, Nicaragua, Pakistan, Peru, Philippines, Venezuela, Austria, Cyprus, Denmark, European Union (EU), Finland, France, Germany, Greece, Ireland, Italy, Malta, Morocco, Netherlands, Norway, Poland, Portugal, Slovenia, Spain, Sweden, and Thailand. The cluster at 6.95 E-03 mg/kg-day is made up of 17 IMDLs computed from Algeria, Angola, Bangladesh, Barbados, Bermuda, Belgium, Bulgaria, Estonia, Hong Kong, French West Indies, Iceland, Latvia, Luxembourg, Romania, Switzerland, United Arab Emirates, and Ukraine. The cluster at 8.57 E-04 mg/kg-day is made up of 21 IMDLs computed from Argentina, Tanzania, Albania, Antigua and Barbuda, Belize, Bhutan, Fiji, India, Kazakhstan, Kiribati, Kuwait, Labia, Nauru, Russia, Rwanda, St. Lucia, Syrian Arab, Tonga, Tuvalu, Uganda, and U.S..

Only four 2,4-D IMDLs are above the arithmetic mean (2.31 E-02 mg/kg-day) because it is skewed by some extreme values such as 8.66 E-01 mg/kg-day at the high end of the distribution. On the other hand, the median and geometric mean (6.94 E-03 and 2.14 E-03 mg/kg-day respectively) are better measures of vales central tendency. Table 3 provides the statistics summary of 2,4-D IMDLs.

There are 13 (9.0% of the total) 2,4-D IMDLs exceeding the 2,4-D ADI which is equal to 0.01 mg/kg-day [21]. Although the rest of IMDLs seem appropriate, it is hard to suggest that many worldwide jurisdictions have provided appropriate 2,4-D PSVs because only jurisdictions from Mexico, Honduras, Pakistan, Peru, and Philippines regulated 2,4-D PSVs in soil, water, and agricultural commodity.

**Table 3.** 2,4-D IMDLs Statistic Summary.

	*Total exposures*	*Three exposures*	*Two exposures*	*One exposure*
Number of IMDLs	145	17	53	75
µ (mg/kg-day)	2.31E-02	1.12E-01	2.31E-02	3.07E-03
µ_G_ (mg/kg-day)	2.14E-03	1.65E-02	5.37E-03	7.04E-04
µ_L_	−2.67E+00	−1.78E+00	−2.27E+00	−3.15E+00
σ_L_	1.16E+00	6.73E-01	7.40E-01	1.26E+00
σ (mg/kg-day)	1.23E-01	2.83E-01	1.18E-01	3.03E-03
Max IMDL (mg/kg-day)	8.66E-01	8.64E-01	8.66E-01	8.06E-03
Min IMDL (mg/kg-day)	1.73E-07	5.69E-03	3.03E-06	1.73E-07
Orders of magnitude variation	6.70E+00	2.18E+00	5.46E+00	4.67E+00
m (mg/kg-day)	6.95E-03	1.08E-02	7.18E-03	8.57E-04

### 4.2. Chlorpyrifos IMDL Analysis

**Figure 2.**
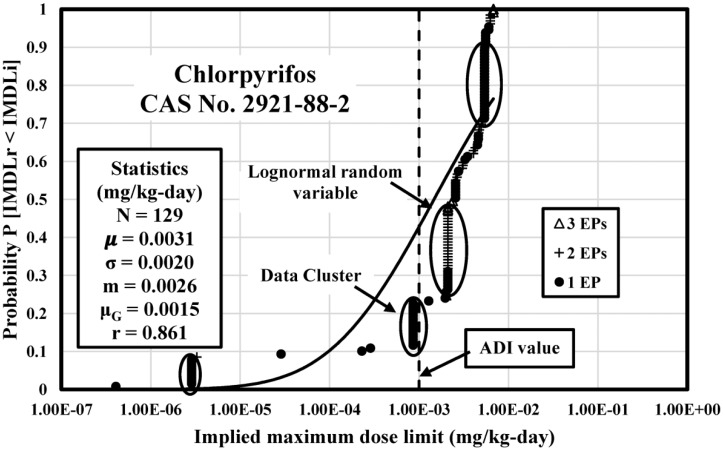
Empirical CDF of Chlorpyrifos IMDLs Computed from Soil, Water, and Agricultural Commodity PSVs Compared to a Theoretical Lognormal CDF with Identical Statistics.

Figure 2 illustrates the CDF of 129 IMDLs calculated from Chlorpyrifos major exposures and compared with a theoretical lognormal CDF calibrated with the computed mean and standard deviation statistics. The IMDLs of Chlorpyrifos range from 4.06 E-07 (Moldova) to 6.77 E-03 mg/kg-day (New Zealand) with 4.22 orders of magnitude. The Pearson correlation coefficient is 0.861, which suggests values are well dispersed over this span. The CDF is skewed by four IMDLs clusters.

The cluster at 2.86E-06 mg/kg-day is made up of 9 IMDLs from Andorra, Bolivia, Bulgaria, Estonia, Gambia, Labia, Liechtenstein, Ukraine, and Vanuatu. The cluster at 8.57 E-04 mg/kg-day is made up of 14 IMDLs from Antigua and Barbuda, Belize, Bhutan, Fiji, Labia, Kiribati, Kuwait, Nauru, Qatar, Tonga, Tuvalu, Uganda, and St. Lucia. The cluster at 2.09 E-03 – 2.10 E-03 mg/kg-day is made up of 29 IMDLs from Austria, Belgium, Bulgaria, French West Indies, Cyprus, Czech Republic, Denmark, EU, Finland, France, Germany, Greece, Iceland, Latvia, Luxembourg, Ireland, Italy, Malta, Netherlands, Norway, Poland, Portugal, Romania, Slovenia, Slovakia, Spain, and United Kingdom. The cluster at 5.38 E-03 – 5.42 E-03 mg/kg-day is made up of 26 IMDLs from Algeria, Angola, Bangladesh, Barbados, Bermuda, Cambodia, Colombia, Costa Rica, Cuba, Dominican Republic, Honduras, El Salvador, Guatemala, Jamaica, Jordan, Kenya, Lebanon, Netherlands Antilles, Nicaragua, Pakistan, Panama, Philippines, Saudi Arabia, Trinidad and Tobago, Tunisia, and Venezuela.

There are 100 (77.5% of the total) Chlorpyrifos IMDLs above the ADI which is 0.001 mg/kg-day [22], which suggests that Chlorpyrifos PSVs from most worldwide jurisdiction can hardly protect human health. For the rest of Chlorpyrifos IMDLs which are below the ADI, none of them account for all major pesticide exposures. Among the 129 Chlorpyrifos IMDLs, only seven of them were computed from three exposures. Table 4 provides the statistics summary of Chlorpyrifos IMDLs.

**Table 4.** Chlorpyrifos IMDLs Statistic Summary.

	*Total exposures*	*Three exposures*	*Two exposures*	*One exposure*
Number of IMDLs	129	7	41	81
µ (mg/kg-day)	3.07E-03	3.48E-03	3.19E-03	2.97E-03
µ_G_ (mg/kg-day)	1.47E-03	3.00E-03	2.53E-03	1.05E-03
µ_L_	−2.83E+00	−2.52E+00	−2.60E+00	−2.98E+00
σ_L_	9.21E-01	2.42E-01	4.96E-01	1.08E+00
σ (mg/kg-day)	2.01E-03	2.24E-03	1.55E-03	2.20E-03
Max IMDL (mg/kg-day)	6.77E-03	6.77E-03	6.24E-03	6.01E-03
Min IMDL (mg/kg-day)	4.06E-07	2.00E-03	3.26E-06	4.06E-07
Orders of magnitude variation	4.22E+00	5.29E-01	3.28E+00	4.17E+00
m (mg/kg-day)	2.55E-03	2.30E-03	2.10E-03	2.55E-03

### 4.3. Diazinon IMDL Analysis

**Figure 3.**
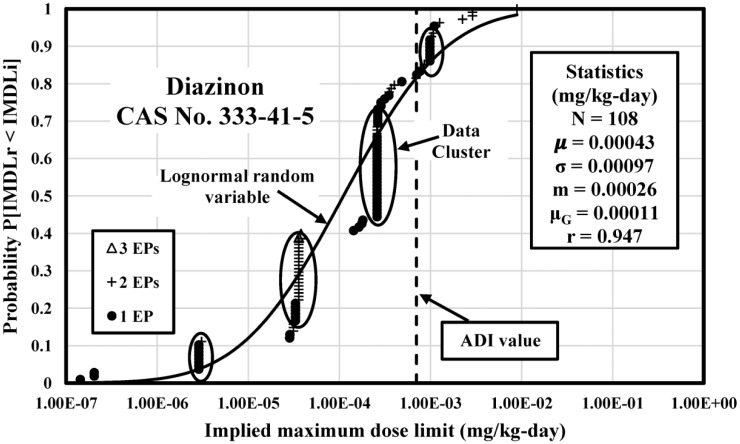
Empirical CDF of Diazinon IMDLs Computed from Soil, Water, and Agricultural Commodity PSVs Compared to a Theoretical Lognormal CDF with Identical Statistics.

Figure 3 illustrates the CDF of 108 IMDLs calculated from Diazinon major exposures and compared with a theoretical lognormal CDF calibrated with the computed mean and standard deviation statistics. The IMDLs of Diazinon range from 1.43 E-07 (Iraq) to 8.90 E-03 mg/kg-day (Russia) with 4.79 orders of magnitude. The Pearson correlation coefficient is 0.947, which suggests values are well dispersed over this span. The CDF is skewed by four IMDLs clusters.

The data cluster at 2.86 E-06 mg/kg-day is made up of 8 IMDLs from Andorra, Bolivia, Bulgaria, Estonia, Labia, Liechtenstein, Ukraine, and Vanuatu. The cluster at 3.58 E-05 mg/kg-day is made up of 18 IMDLs from the Austria, Cyprus, Czech Republic, Denmark, EU, France, Germany, Greek, Ireland, Italy, Lithuania, Malta, Netherlands, Poland, Portugal, Slovenia, Sweden, and Switzerland. The cluster at 2.59 E-04 – 2.63 E-04 mg/kg-day is made up of 32 IMDLs from Algeria, Angola, Bangladesh, Barbados, Cambodia, China, Chile, Costa Rica, Egypt, Ecuador, El Salvador, Guatemala, Jamaica, Jordan, Kenya, Lebanon, Morocco, Netherlands Antilles, Nicaragua, Pakistan, Panama, Peru, Philippines, South Africa, Thailand, Trinidad and Tobago, Tunisia, United Arab Emirates, Venezuela, Vietnam, and WHO. The cluster at 9.84 E-04 – 1.11 E-03 mg/kg-day is made up of 11 IMDLs from Bahrain, Brunei, Hong Kong, South Korea, New Zealand, Kuwait, Oman, Qatar, Saudi Arabia, and Singapore.

Only 22 Diazinon IMDLs are above the arithmetic mean which is 4.26 E-04 mg/kg-day because it is skewed by some extreme values such as 8.90 E-03 mg/kg-day at the high end of the distribution. The median and geometric mean (2.59 E-04 and 1.11 E-04 mg/kg-day, respectively) are probably better measures of vales central tendency. Among the 108 Diazinon IMDLs, only two of them were computed from three exposures. Table 5 provides statistics summary of Diazinon IMDLs.

**Table 5.** Diazinon IMDLs Statistic Summary.

	*Total exposures*	*Three exposures*	*Two exposures*	*One exposure*
Number of IMDLs	108	2	39	67
µ (mg/kg-day)	4.26E-04	3.68E-05	6.75E-04	2.93E-04
µ_G_ (mg/kg-day)	1.11E-04	3.68E-05	1.34E-04	1.02E-04
µ_L_	−3.96E+00	−4.43E+00	−3.87E+00	−3.99E+00
σ_L_	8.90E-01	1.68E-02	7.97E-01	9.51E-01
σ (mg/kg-day)	9.69E-04	1.42E-06	1.54E-03	3.02E-04
Max IMDL (mg/kg-day)	8.90E-03	3.78E-05	8.90E-03	1.10E-03
Min IMDL (mg/kg-day)	1.43E-07	3.58E-05	3.06E-06	1.43E-07
Orders of magnitude variation	4.79E+00	2.38E-02	3.46E+00	3.89E+00
m (mg/kg-day)	2.59E-04	3.68E-05	3.58E-05	2.59E-04

There are 20 (18.5% of the total) Diazinon IMDLs above the ADI which is 0.002 mg/kg-day [23] indicating that these Diazinon PSVs from worldwide jurisdiction can hardly protect human health. For the rest of Diazinon IMDLs which are below the ADI, only jurisdictions from Slovakia and the Czech Republic regulated Diazinon PSVs in major exposures.

## 5. Summary and Conclusions

Table 6 provides statistics information for these selected pesticides (Bromomethane and Toxaphene were omitted due to few jurisdictions regulated PSVs for them). Vietnam contributes ten maximum IMDLs not only because Vietnam provided PSVs in three major exposures but also Vietnam regulated relatively large pesticide drinking water MCLs. Russia and Croatia contribute three maximum IMDLs. For most nations with minimum IMDLs, they only regulated PSVs in one exposure pathway. For example, Moldova contributes four minimum IMDLs which computed from soil RGVs, and Iraq contributes three minimum IMDLs computed from drinking water MCLs only.

The weighted average Pearson correlation coefficient of selected pesticides IMDLs is 0.926. For some pesticides such as Dieldrin, the correlation coefficient is 0.981. The weighted average order of variance of IMDLs is 6.09. Endosulfan IMDL values have the largest span of 8.29 order of magnitude. It suggests that in general, the IMDLs of selected pesticides are well dispersed over data spans, and worldwide jurisdictions lack the agreement on PSVs regulations in major exposures.

Only 105 IMDLs (4% of the total number of the selected pesticides) were computed from three major exposures. Most worldwide jurisdictions regulated selected pesticides in either two exposures or one exposure. As those are largely used pesticides and they can move and be transported to the soil, water, air, and biomass. It is necessary for worldwide jurisdictions to regulate PSVs in all major exposures. Glyphosate is top used pesticides over the world, however, only four Glyphosate IMDLs were computed from PSVs in soil, water, and agricultural commodity. Although the use of DDT has been banned, it can still be detected in soil, water, and food because of the wide application in the past.

There are 100 Chlorpyrifos IMDLs (77.5% of the total) above the ADI, however, only seven IMDLs were computed from major exposures, indicating that jurisdictions haven't provide safe Chlorpyrifos standard values even in one of the major exposure pathways. Although all IMDLs of Endosulfan are below the ADI value, none of them account for all major human exposures. Above all, it suggests that PSVs in all major exposure pathways should be regulated and comprehensive regulations of PSVs are necessary from human health point of view.

**Table 6.** Statistic Summary for Selected Pesticides.

*Pesticide*	*CAS No.*	*Pearson correlation coefficient*	*Log orders of variance*	*Max IMDL (mg/kg-day)*	*Min IMDL (mg/kg-day)*	*Total number of IMDLs*	*Number of IMDLs computed from three exposures*	*Number of IMDLs above ADI*
2,4-D	94-75-7	0.881	6.70	8.66 E-01, Viet Nam	1.73 E-07, Armenia *	145	17	13 (9.0%)
Aldicarb	116-06-3	0.977	5.00	2.86 E-01, Viet Nam	2.86 E-06, Andorra *	121	5	3 (2.5%)
Aldrin	309-00-2	0.824	5.15	2.86 E-03, Croatia	2.03 E-08, Italy	147	0	22 (9.0%)
Atrazine	1912-24-9	0.917	6.75	5.70 E-02, Viet Nam	1.02 E-08, Ecuador	125	22	2 (1.6%)
Chlordane	57-74-9	0.923	8.06	5.71 E-03, Viet Nam *	5.01 E-11, Serbia	131	5	9 (6.9%)
Chlorothalonil	1897-45-6	0.925	3.51	9.22 E-03, Australia	2.86 E-06, Andorra *	105	2	0 (0.0%)
Chlorpyriphos	2921-88-2	0.861	4.22	6.77 E-03, New Zealand	4.06 E-07, Moldova	129	7	100 (77.5%)
DDT	50-29-3	0.979	6.55	5.71 E-02, Viet Nam	1.62 E-08, Montenegro	161	0	25 (15.5%)
Diazinon	333-41-5	0.947	4.79	8.90 E-03, Russia	1.43 E-07, Iraq	108	2	20 (18.5%)
Dicamba	1918-00-9	0.713	4.34	1.10 E-02, Russia	5.08 E-07, Uzbekistan	105	2	0 (0.0%)
Dieldrin	60-57-1	0.981	5.15	2.86 E-03, Croatia	2.03 E-08, Italy	140	0	20 (14.2%)
Diuron	330-54-1	0.946	4.45	2.86 E-02, Russia	1.02 E-06, Moldova *	75	2	11 (11.4%)
Endosulfan	115-29-7	0.941	8.29	3.94 E-03, Argentina	2.03 E-11, Serbia *	76	0	0 (0.0%)
Endrin	72-20-8	0.915	7.55	2.87 E-03, Hungary	8.13 E-11, Serbia	102	0	6 (5.9%)
Glyphosate	1071-83-6	0.854	6.11	1.86 E-01, Guatemala	1.43 E-07, Iraq	115	4	42 (27.1%)
Heptachlor	76-44-8	0.981	6.30	2.86 E-03, Croatia	1.42 E-09, Singapore	113	8	18 (15.9%)
Lindane	58-89-9	0.969	7.53	5.71 E-02, Viet Nam	1.67 E-09, Bulgaria	153	4	14 (9.2%)
Malathion	121-75-5	0.688	5.91	1.17 E-01, Viet Nam	1.43 E-07, Iraq	111	2	94 (84.7%)
Mancozeb	8081-01-7	0.719	5.11	2.63 E-02, U.S.	2.03 E-07, Belarus *	105	2	0 (0.0%)
MCPA	94-74-6	0.917	5.85	5.78 E-02, Viet Nam	8.13 E-08, Belarus	126	12	39 (69.0%)
Metolachlor	51218-45-2	0.921	5.78	2.44 E-02, Bahamas	4.06 E-08, Georgia	77	3	0 (0.0%)
Pentachlorophenol	87-86-5	0.970	5.94	2.57 E-01, Viet Nam	2.94 E-07, Moldova	130	4	2 (1.5%)
Trifluralin	1582-09-8	0.897	6.45	5.71 E-01, Viet Nam	2.03 E-07, Moldova	98	2	1 (1.0%)

* The values are also shared by other nation.

## Figures and Tables

**Table S1:** Worldwide pesticide soil regulatory jurisdictions references and dates accessed

Jurisdictions	Sources	Languages	Websites References
*Multinational organizations Multinational organizations*			
**East Africa Community** (Kenya, Uganda, Tanzania, Rwanda, Burundi)	East Africa Community (2011)	English	East Africa Community, 2011. Thematic Area 2 Environmental Regulations and Standards. http://www.eac.int/infrastructure/ (accessed 09.09.13).
**European Union**	European Union (2010)	English	European Union, 2010. Regulations Commission Regulation (EU) No 756/2010. http://eur-lex.europa.eu/LexUriServ/LexUriServ.do?uri=OJ:L:2010:223:0020:0028:EN:PDF (accessed 09.09.13).
**World Health Organization** (2002)	World Health Organization (2002)	English	World Health Organization, 2002. Developing Human Health-related Chemical Guidelines for Reclaimed Water and Sewage Sludge Applications in Agriculture 2002. http://www.who.int/en/http://www.opaci.org.py/biblioteca/Servicio_Sanitario/Guia_Calidad_Agua/CD_GuiasOMS/Biblioteca/AguasResiduales/gwwuchemicals.pdf (accessed 08.09.13).
*United Nations member states United Nations member states*			
Principality of **Andorra**	Andorra Official Gazette (2010)	Catalan	Andorra Official Gazette, 2010. Decree approving the Regulation of contaminated soil (in Catalan). http://www.bopa.ad/bopa.nsf/c56341fced070c89c12566c700571ddd/54e68267f52e69b7c12576cf00372375?OpenDocument (accessed 16.07.13).
Republic of **Armenia**	Armenia Minister Of Health (2011)	Armenian	Armenia Minister of Health, 2011. Land and the Rules and Hygiene Requirements N2.1.7.003-10 (in Armenian). www.awhhe.am/downloads/eu_project_presentations/POPs_in_soil.doc (accessed 16.07.13).
Commonwealth of **Australia**	Australia National Environmental Protection Council (1999)	English	Australia National Environmental Protection Council, 1999. National Environment Protection (Assessment of Site Contamination) Measure 1999. http://www.comlaw.gov.au/Details/F2013C00288/Html/Volume_2 (accessed 30.07.13).
(Australia) **Australian Capital Territory**	Australia National Environmental Protection Council (1999)	English	Australia National Environmental Protection Council, 1999. National Environment Protection (Assessment of Site Contamination) Measure 1999. http://www.comlaw.gov.au/Details/F2013C00288/Html/Volume_2 (accessed 30.07.13).
(Australia) State of **Tasmania**	Tasmania Environmental Protection Authority (2012)	English	Tasmania Environmental Protection Authority, 2012. Classification and Management of Contaminated Soil for Disposal. http://epa.tas.gov.au/documents/ib105_classification_and_management_of_contaminated_soil_2012.pdf (accessed 29.07.13).
	Australia National Environmental Protection Council (1999)	English	Australia National Environmental Protection Council, 1999. National Environment Protection (Assessment of Site Contamination) Measure 1999. http://www.comlaw.gov.au/Details/F2013C00288/Html/Volume_2 (accessed 30.07.13).
(Australia) State of **New South Wales**	New South Wales Department of Environment and Conservation (2006)	English	New South Wales Department of Environment and Conservation, 2006. Guidelines for the NSW Site Auditor Scheme (2nd edition). http://www.epa.nsw.gov.au/resources/clm/auditorglines06121.pdf (accessed 29.07.13).
(Australia) State of **Northern Australia**	Australia National Environmental Protection Council (1999)	English	Australia National Environmental Protection Council, 1999. National Environment Protection (Assessment of Site Contamination) Measure 1999. http://www.comlaw.gov.au/Details/F2013C00288/Html/Volume_2 (accessed 30.07.13).
(Australia) State of **Queensland**	Australia National Environmental Protection Council (1999)	English	Australia National Environmental Protection Council, 1999. National Environment Protection (Assessment of Site Contamination) Measure 1999. http://www.comlaw.gov.au/Details/F2013C00288/Html/Volume_2 (accessed 30.07.13).
(Australia) State of **Victoria**	Victoria Environmental Protection Authority (2002)	English	Victoria Environmental Protection Authority, 2002. Victoria Government Gazette. http://www.gazette.vic.gov.au/ (accessed 12.08.13).
	Australia National Environmental Protection Council (1999)	English	Australia National Environmental Protection Council, 1999. National Environment Protection (Assessment of Site Contamination) Measure 1999. http://www.comlaw.gov.au/Details/F2013C00288/Html/Volume_2 (accessed 30.07.13).
(Australia) State of **South Australia**	Australia National Environmental Protection Council (1999)	English	Australia National Environmental Protection Council, 1999. National Environment Protection (Assessment of Site Contamination) Measure 1999. http://www.comlaw.gov.au/Details/F2013C00288/Html/Volume_2 (accessed 30.07.13).
	South Australia Environment Protection Authority (2006)	English	South Australia Environment Protection Authority, 2006. EPA Guidelines for Environmental management of on-site remediation. http://www.epa.sa.gov.au/xstd_files/Site%20contamination/Guideline/guide_remediation.pdf (accessed 23.07.13).
(Australia) State of **Western Australia**	Western Australia Department of Environment And Conservation (2010)	English	Western Australia Department of Environment and Conservation, 2010. Assessment Levels for Soil, Sediment and Water. http://www.dec.wa.gov.au/search.html?searchword=assessment%20levels%20for%20soil,%20sediment%20and&searchphrase=all (accessed 29.07.13).
Commonwealth of the **Bahamas**	Bahamas Ministry of Works And Transport (2008)	English	Bahamas Ministry of Works and Transport, 2008. SUPPLEMENTARYFINANCINGFORNEWPROVIDENCE TRANSPORTPROGRAM-BAHAMAS (BH-L1024). http://idbdocs.iadb.org/wsdocs/getdocument.aspx?docnum=1415036 (accessed 14.08.13).
Republic of **Belarus**	Belarus Ministry of Health (2004)	Belarusian	Belarus Ministry of Health, 2004. Hygienic standards 2.1.7.12-1-2004 “The list of maximum permissible concentration (MPC) and the approximate permissible concentrations (APC) of chemical substances in soil” (in Belarusian). http://minzdrav.gov.by/ru/static/acts/tehnicheskie/teksty/pochva (accessed 05.08.13).
Federative Republic of **Brazil**	Brazil Ministry of The Environment (2009)	Portuguese	Brazil Ministry of the Environment, 2009. National Council on the Environment Resolution No. 420, 28 December 2009 (in Portuguese). http://www.mma.gov.br/port/conama/legiabre.cfm?codlegi=620 (accessed 02.08.13).
(Brazil) State of **San Paolo**	Environmental Company of Sao Paolo (2005)	Portuguese	Environmental Company of Sao Paolo, 2005. Guiding Values for Soils and Groundwater in the State of San Paulo (in Portuguese). http://www.cetesb.sp.gov.br/Solo/relatorios/tabela_valores_2005.pdf (accessed 02.08.13).
Republic of **Bulgaria**	Bulgaria Ministry of Environment And Water (2008)	Bulgarian	Bulgaria Ministry of Environment and Water, 2008. Ordinance № 3 from 1 August 2008, limit values of harmful substances in the soil (in Bulgarian). http://www.moew.government.bg/?show=top&cid=388 (accessed 12.08.13).
**Canada**	Canadian Council of Ministers of The Environment (CCME) (2011)	English	Canadian Council of Ministers of the Environment (CCME), 2011. Soil Quality Guidelines for the Protection of Environmental and Human Health. http://st-ts.ccme.ca/ (accessed 14.08.13).
(Canada) Province of **Alberta**	Alberta Environment (2010)	English	Alberta Environment, 2010. Alberta Tier 1 Soil and Groundwater Remediation Guidelines (2010). http://www.environment.alberta.ca/01058.html (accessed 15.08.13).
(Canada) Province of **British Columbia**	British Columbia Regulations (2013)	English	British Columbia Regulations, 2013. Schedule 5, Environmental Management Act CONTAMINATED SITES REGULATION. http://www.bclaws.ca/EPLibraries/bclaws_new/document/LOC/freeside/--%20E%20--/Environmental%20Management%20Act%20SBC%202003%20c.%2053/05_Regulations/21_375_96%20Contaminated%20Sites%20Regulation/375_96_07.xml#Schedule5 (accessed 15.08.13).
	British Columbia Regulations (2013)	English	British Columbia Regulations, 2013. Schedule 7, Environmental Management Act CONTAMINATED SITES REGULATION. http://www.bclaws.ca/EPLibraries/bclaws_new/document/LOC/freeside/--%20E%20--/Environmental%20Management%20Act%20SBC%202003%20c.%2053/05_Regulations/21_375_96%20Contaminated%20Sites%20Regulation/375_96_09.xml#Schedule7 (accessed 15.08.13).
	British Columbia Regulations (2013)	English	British Columbia Regulations, 2013. Schedule 10, Environmental Management Act CONTAMINATED SITES REGULATION. http://www.bclaws.ca/EPLibraries/bclaws_new/document/LOC/freeside/--%20E%20--/Environmental%20Management%20Act%20SBC%202003%20c.%2053/05_Regulations/21_375_96%20Contaminated%20Sites%20Regulation/375_96_12.xml#Schedule10 (accessed 15.08.13).
(Canada) Province of **Manitoba**	Manitoba Conservation (2011), CCME (2011)	English	Manitoba Conservation, 2011. Environmental Services Criteria/Standards and Guidelines. http://www.gov.mb.ca/conservation/envprograms/contams/standards/index.html (accessed 15.08.13).
(Canada) Province of **Newfoundland and Labrador**	Environment and Conservation, Government of Newfoundland and Labrador (2005)	English	Environment and Conservation, Government of Newfoundland and Labrador, 2005. Guidance Document for the Management of Impacted Sites. http://www.env.gov.nl.ca/env/env_protection/ics/guidancedocv.1.01external.pdf (accessed 15.08.13).
(Canada) **Northwest Territories**	Northwest Territories Department of Environment and Natural Resources (2003)	English	Northwest Territories Department of Environment and Natural Resources, 2003. Environmental Guideline for Contaminated Site Remediation. http://www.enr.gov.nt.ca/_live/documents/content/siteremediation.pdf (accessed 19.08.13).
Canada **Nova Scotia**	Nova Scotia Environment (2013)	English	Nova Scotia Environment, 2013. Table 1A Tier 1 Environmental Quality Standards for Soil at a Potable Site. http://www.novascotia.ca/nse/contaminatedsites/docs/Table_1A_Tier1_EQS_for_Soil_Potable_Site.pdf (accessed 15.08.13).
			Nova Scotia Environment, 2013. Table 1B Tier 1 Environmental Quality Standards for Soil at a Non-Potable Site. http://www.novascotia.ca/nse/contaminatedsites/docs/Table_1B_Tier1_EQS_for_Soil_Non-Potable_Site.pdf (accessed 15.08.13).
(Canada) **Nunavut**	Department of Environment, Government of Nunavut (2009)	English	Department of Environment, Government of Nunavut, 2009. Environmental Guideline for Contaminated Site Remediation. http://env.gov.nu.ca/sites/default/files/Guideline%20Contaminated%20Site%20Remediation.pdf (accessed 19.08.13).
(Canada) Province of **Ontario**	Ontario Ministry of The Environment (2011)	English	Ontario Ministry of the Environment, 2011. Soil, Ground Water and Sediment Standards for Use Under Part XV.1 of the Ontario Protection Act.PIBS# 7382e01. http://www.ene.gov.on.ca/environment/en/subject/brownfields/STDPROD_075745.html (accessed 19.08.13).
(Canada) Province of **Prince Edward Island**	Prince Edward Island Environment, Energy and Forestry (2010)	English	Prince Edward Island Environment, Energy and Forestry, 2010. Chapter E-9 Environmental Protection Act Contaminated Sites Registry Regulations. http://www.gov.pe.ca/law/regulations/pdf/E&09-03.pdf (accessed 19.08.13).
(Canada) Province of **Quebec**	Quebec Ministry of Sustainable Development, Environment and Parks (1998)	French	Quebec Ministry of Sustainable Development, Environment and Parks, 1998. Appendix 2: Generic criteria for soils and groundwater, Policy of soil protection and remediation of contaminated land (in French). http://www.mddep.gouv.qc.ca/sol/terrains/politique/annexe_2_tableau_1.htm (accessed 19.08.13).
(Canada) **Yukon**	Yukon Regulations (2002)	English	Yukon Regulations, 2002. O.I.C. 2002/171 Environment Act. http://www.gov.yk.ca/legislation/regs/oic2002_171.pdf (accessed 19.08.13).
People's Republic of **China**	Peoples Republic of China (1995)	Chinese	Peoples Republic of China, 1995. Environmental quality standard for soils (GB 15618-1995 1996-03-01) (in Chinese). http://kjs.mep.gov.cn/hjbhbz/bzwb/trhj/trhjzlbz/199603/t19960301_82028.htm (accessed 20.08.13).
	People's Republic of China Ministry of Environmental Protection (2006)	Chinese	People's Republic of China Ministry of Environmental Protection, 2006. National Standard for edible agriculture product (in Chinese). http://kjs.mep.gov.cn/hjbhbz/bzwb/trhj/trhjzlbz/200412/t20041229_63466.htm (accessed 20.08.13).
Peoples Republic of China (**Beijing**)	Beijing Municipal Environmental Protection Bureau (2011)	Chinese	Beijing Municipal Environmental Protection Bureau, 2011. Environmental Risk Assessment Screening venues soil values (in Chinese). http://www.bjepb.gov.cn/ (accessed 20.08.13).
Republic of **Costa Rica**	Ministry of Health (2011)	Spanish	Ministry of Health, 2011. No.-S-MINAET-MAG The President of the Republic Health Minister, Minister Of Environment, Energy and Telecommunications, and the Minister of Agriculture and Livestock (in Spanish). http://www.amcham.co.cr/newsletters/201103/contaminantes.pdf (accessed 21.08.13).
(**Croatia**) Agricultural Uni. Of Zagreb	Agricultural University of Zagreb (2008)	Croatian	Agriculture University of Zagreb, 2008. Preparation of Croatian Soil Monitoring Pilot Project (LIFE05 TCY/CRO/000105 Final Report) (in Croatian). http://www.azo.hr/Projects05 (accessed 21.08.13).
**Czech** Republic	Czech Republic Ministry of The Environment (1994)	English	Czech Republic Ministry of Environment, 1994. Decree 13/1994 of the Ministry of the Environment of the Czech Republic Specifying Some Details of Agricultural Land Resources Protection. http://faolex.fao.org/docs/pdf/cze62400E.pdf (accessed 22.08.13).
	European Commission (2007)	English	European Commission, 2008. Priority Substances and Certain other Pollutants (According to AnnexII of theDirective2008/105/EC). http://ec.europa.eu/environment/water/water-framework/priority_substances.htm (accessed 22.08.13).
Kingdom of **Denmark**	Danish Environmental Protection Agency (2010)	Danish	Danish Environmental Protection Agency, 2010. List of quality criteria in the contaminated soil and quality of drinking water (in Danish). http://www.mst.dk/NR/rdonlyres/0AB0AF23-4BD6-4901-BCD9-43F6F6FD6FC1/124958/Kvalitetskriterierjord_og_drikkevandfinaljuniogjul.pdf (accessed 22.08.13).
Republic of **Ecuador**	Ecuador Ministry of the Environment (2004)	Spanish	Ecuador Ministry of Environment, 2004. Environmental Quality Standard of Soil Resources and Criteria for Contaminated Soil (in Spanish). http://faolex.fao.org/docs/pdf/ecu112181.pdf (accessed 23.08.13).
Republic of **Estonia**	Estonia Ministry of the Environment (2004)	Estonian	Estonia Ministry of the Environment, 2004. In Soil and Groundwater Concentration Limits for of Hazardous Substances (No 12, Adopted 04/02/2004 RTL 2004, 40,662) (in Estonian). https://www.riigiteataja.ee/akt/961568 (accessed 23.08.13).
Republic of **Finland**	Finland Ministry Of The Environment (2007)	Finish	Finland Ministry of the Environment, 2007. Government Regulation of Soil Contamination and Cleaning Needs Assessment (in Finish). http://www.finlex.fi/data/sdliite/liite/5382.pdf (accessed 22.08.13).
French Republic (**France**)	European Commission (2007)	English	European Union, 2010. Regulations Commission Regulation (EU) No 756/2010. http://eur-lex.europa.eu/LexUriServ/LexUriServ.do?uri=OJ:L:2010:223:0020:0028:EN:PDF (accessed 09.09.13).
**Georgia**	Georgia Minister of Environment and Minister of Natural Resources (2006)	Georgian	Georgia Ministry of Environment and Minister of Natural Resources, 2006. Decree N538 e For the Calculation of Damage to the Environment. Appendix 4-Pollution of Land Quality Assessment Table (in Georgian). http://www.cenn.org/wssl/programs/zianis_daangariSebis_metodika_Methodology_Environmental_Damage.pdf (accessed 03.09.13).
	Minister of Health, Labor and Social Affairs (2001)	Georgian	Minister of Health, labor and social affairs, 2001. Environmental concentrations of pesticides approved hygiene standards (in Georgian). http://medportal.ge/mohsrv/lawfiles/781.htm (accessed 03.09.13).
Federal Republic of **Germany**	German Federal Ministry of the Environment, Nature Conservation and Nuclear Safety (1999)	German	German Federal Ministry of the Environment, Nature Conservation and Nuclear Safety, 1999. Federal Soil Protection and Contaminated Sites Regulation (BBODSCHG) (in German). http://www.bmu.de/fileadmin/bmu-import/files/pdfs/allgemein/application/pdf/bbodschv_uk.pdf (accessed 23.08.13).
**Hungary**	Hungary Ministry of the Environment (2000)	Hungarian	Hungary Ministry of the Environment, 2000. (June 2) Ministry of Environment, Ministry of Health, Ministry of Agriculture, Ministry of Transport co-regulation of groundwater limit values ??and quality necessary to protect the geological (in Hungarian). http://www.kvvm.hu/szakmai/karmentes/jogszab/jogszab02/tartalom2.htm (accessed 26.08.13).
Republic of **Italy**	Italy National Institute of Health (2006)	Italian	Italy National Institute of Health, 2006. Official Gazette no. 293 15-12-1999 (in Italian). http://gazzette.comune.jesi.an.it/293-99/supll218.htm (accessed 26.08.13).
(Italy) **Lombardi** Region	Tazzioli (1999)	Italian	Tazzioli, R., 1999. The Quality Standards Applicable for the Protection of Soil Pollution (in Italian). http://www.sindar.it/Editoriale/Allegati/Argomenti/suoli1.pdf (accessed 25.08.10).
(Italy) **Piedmont** Region	Tazzioli (1999)	Italian	Tazzioli, R., 1999. The Quality Standards Applicable for the Protection of Soil Pollution (in Italian). http://www.sindar.it/Editoriale/Allegati/Argomenti/suoli1.pdf (accessed 25.08.10).
(Italy) **Emili Romana** Region	Tazzioli (1999)	Italian	Tazzioli, R., 1999. The Quality Standards Applicable for the Protection of Soil Pollution (in Italian). http://www.sindar.it/Editoriale/Allegati/Argomenti/suoli1.pdf (accessed 25.08.10).
(Italy) **Liguria** Region	Tazzioli (1999)	Italian	Tazzioli, R., 1999. The Quality Standards Applicable for the Protection of Soil Pollution (in Italian). http://www.sindar.it/Editoriale/Allegati/Argomenti/suoli1.pdf (accessed 25.08.10).
Republic of **Latvia**	Latvia Cabinet of Ministers (2005)	Latvian	Latvia Cabinet of Ministers, 2005. Terms of soil and soil quality standards (in Latvian). http://likumi.lv/doc.php?id=120072 (accessed 27.08.13).
Republic of **Lithuania**	Lithuania Ministry of the Environment (2008)	Lithuanian	Contaminated Sites Management Environmental Protection (April 30, 2008) (in Lithuanian). http://tar.tic.lt/Default.aspx?id¼2&item¼results&aktoid¼43AD7376-25A2-47B1-9378-554EE563D95B (accessed 27.08.13).
**Malaysia**	Malaysia Environment Protection Department (2009)	English	Malaysia Department of Environment, 2009. Contaminated Land Management and Control Guidelines No. 1: Malaysian Recommended Site Screening Levels for Contaminated Land. http://www.doe.gov.my/portal/wp-content/uploads/Contaminated-Land-Management-and-Control-Guidelines-No-1_Malaysian-Recommended-Site-Screening-Levels-for-Contaminated-Land.pdf (accessed 28.08.13).
Republic of **Moldova**	Moldova Ministry of Ecology and Natural Resources (2004)	Romanian	Moldova Ministry of Ecology and Natural Resources, 2004. NOTE Nr. 383 of 08.08.2004 Instructions injury assessment of soil resources (in Romanian). http://lex.justice.md/index.php?action=view&view=doc&lang=1&id=310719 (accessed 30.08.13).
Kingdom of the **Netherlands**	Netherlands National Institute for Public Health and the Environment (2006)	English	Netherlands National Institute for Public Health and the Environment (2006). Preliminary draft of the Soil Quality Regulation. http://www.rwsleefomgeving.nl/publish/pages/92483/soil_quality_regulation_first_notification_24_311345.pdf (accessed 02.09.2013).
	Netherlands Ministry of Economic Affairs, Agriculture and Innovation (2006)	English	Netherlands National Institute for Public Health and Environment, 2001. Technical Evaluation of the Intervention Values for Soil/Sediment and Groundwater, RIVM Report 711701 023. http://www.rivm.nl/bibliotheek/rapporten/711701023.pdf (accessed 01.09.13).
	Netherlands National Institute for Public Health and the Environment (2009)	English	Netherlands National Institute for Public Health and the Environment, 2009. Soil Remediation Circular 2009 (in Dutch). http://www.esdat.com.au/Environmental%20Standards/Dutch/ENGELSE%20versie%20circulaire%20Bodemsanering%202009.pdf (accessed 02.09.2013).
**New Zealand**	New Zealand Ministry of the Environment (2012)	English	New Zealand Ministry of the Environment, 2012. Users' Guide National Environmental Standard for Assessing and Managing Contaminants in Soil to Protect Human Health. http://www.mfe.govt.nz/publications/rma/users-guide-nes-for-assessing-managing-contaminants-in-soil/guide-nes-for-assessing-managing-contaminants-in-soil.pdf (accessed 06.09.13).
	New Zealand Ministry of the Environment	English	New Zealand Ministry of the Environment. Proposed National Environmental Standard for Assessing and Managing Contaminants in Soil. http://www.mfe.govt.nz/publications/rma/proposed-nes-managing-contaminants-in-soil/page8.html (accessed 06.09.13).
	New Zealand Ministry of the Environment (1997)	English	New Zealand Ministry of the Environment, 1997. Health and Environmental Guidelines for Selected Timber Treatment Chemicals. http://www.mfe.govt.nz/publications/hazardous/timber-guide-jun97/timber-guide-jun97.pdf (accessed 06.09.13).
	New Zealand Ministry of the Environment (2006)	English	New Zealand Ministry of the Environment, 2006. Identifying, Investigating and Managing Risks Associated with Former Sheep-dip Sites. http://www.mfe.govt.nz/publications/hazardous/risks-former-sheep-dip-sites-nov06/risks-former-sheep-dip-sites-nov06.pdf (accessed 06.09.13).
	New Zealand Ministry of the Environment (2011)	English	New Zealand Ministry of the Environment, 2011. Methodology for Deriving Standards for Contaminants in Soil to Protect Human Health. http://www.mfe.govt.nz/publications/hazardous/deriving-standards-for-contaminants-in-soil/methodology-for-deriving-standards-for-contaminants-in-soil.pdf (accessed 06.09.13).
	New Zealand Ministry of the Environment (2013)	English	New Zealand Ministry of the Environment, 2013. Environmental Guideline Value (EGV). https://www.mfe.govt.nz/issues/managing-environmental-risks/contaminated-land/managing/egv-database.html (accessed 23.04.14).
(New Zealand) **Auckland City Council**	Cavanagh (2006)	English	Cavanagh, J.E., 2006. Comparison of Soil Guideline Values in New Zealand and Their Derivations. http://ecan.govt.nz/publications/Reports/ComparisonofSoilGuidelineValuesinNewZealand.pdf (accessed 06.09.13).
(New Zealand) **Auckland Regional Council**	Cavanagh (2006)	English	Cavanagh, J.E., 2006. Comparison of Soil Guideline Values in New Zealand and Their Derivations. http://ecan.govt.nz/publications/Reports/ComparisonofSoilGuidelineValuesinNewZealand.pdf (accessed 06.09.13).
(New Zealand) **Bay of Plenty**	Cavanagh (2006)	English	Cavanagh, J.E., 2006. Comparison of Soil Guideline Values in New Zealand and Their Derivations. http://ecan.govt.nz/publications/Reports/ComparisonofSoilGuidelineValuesinNewZealand.pdf (accessed 06.09.13).
(New Zealand) **Hastings District Council**	Cavanagh (2006)	English	Cavanagh, J.E., 2006. Comparison of Soil Guideline Values in New Zealand and Their Derivations. http://ecan.govt.nz/publications/Reports/ComparisonofSoilGuidelineValuesinNewZealand.pdf (accessed 06.09.13).
(New Zealand) **Tasmasn District Council**	Cavanagh (2006)	English	Cavanagh, J.E., 2006. Comparison of Soil Guideline Values in New Zealand and Their Derivations. http://ecan.govt.nz/publications/Reports/ComparisonofSoilGuidelineValuesinNewZealand.pdf (accessed 06.09.13).
(New Zealand) **Waikato Region**	Cavanagh (2006)	English	Cavanagh, J.E., 2006. Comparison of Soil Guideline Values in New Zealand and Their Derivations. http://ecan.govt.nz/publications/Reports/ComparisonofSoilGuidelineValuesinNewZealand.pdf (accessed 06.09.13).
Kingdom of **Norway**	Norwegian Pollution Control Authority (1999)	English	Norwegian Pollution Control Authority, 1999. Guidelines for the risk assessment of contaminated sites. http://www.miljodirektoratet.no/publikasjoner/luft/1691/ta1691.pdf (accessed 05.09.13).
Republic of **Panama**	Panama Ministry of Economy and Finance (2009)	Spanish	Panama Ministry of Economy and Finance, 2009. Executive Order 2-Which Sets the Standard for Soil Environmental Quality for Different Applications (in Spanish). http://www.anam.gob.pa/images/stories/normas_ambientalesdiproca/Decreto_Ejecutivos_No_2.pdf (accessed 05.09.13).
Republic of **Peru**	Peru Ministry of Environment (2013)	Spanish	Peru Ministry of Environment, 2013. Environmental Quality Standards (ECA) (in Spanish). http://www.minam.gob.pe/index.php?option=com_content&view=article&id=102:est (accessed 05.09.13).
Republic of **Poland**	Poland Minister of the Environment (2002)	Polish	Poland Minister of the Environment, 2002. Dz.U.2002.165.1359-On Quality Standards for Soil and Land (4 Oct. 2002) (in Polish). http://www.abc.com.pl/du-akt/-/akt/dz-u-02-165-1359 (accessed 05.09.13).
Republic of **Portugal**	Ontario Ministry of Environment And Energy (1997)	Portuguese and English	Ontario Ministry of Environment and Energy, 1997. Recommendations of the Institute of Waste (in Portuguese). http://ia600409.us.archive.org/9/items/guidelineforusea00ontauoft/guidelineforusea00ontauoft.pdf (accessed 05.09.13).
**Qatar**	Qatar Ministry of Environment (2007)	Arabic	Qatar Ministry of Environment, 2007. Ministry of Environment Implements a Program on Soil Quality in Qatar for Three Years (in Arabic). http://www.moe.gov.qa/English/News/Local/Pages/Ministry-of-Environment-Implements-a-Program-on-Soil-Quality-in-Qatar-for-Three-Years.aspx (accessed 06.09.13).
**Romania**	Romania Ministry of Waters, Forests and Environmental Protection (1997)	Romanian and English	Romanian Ministry of Water Forests and Environmental Protection, 1997. Approving the Regulation on the Assessment of Environmental Pollution. Official Gazette of Romania No. 303bis/06.11.1997 (in Romanian). http://www.moficial.ro/1997/303bis.pdf (accessed 06.12.11).
Russian Federation (**Russia**)	Russian State Construction Code (1997)	Russian	Russian State Construction Code, 1997. P 11-102-97 Engineering and Environmental Surveys for Construction. Appendix B. Criteria for Environmental Assessment of Pollution of Soil and Groundwater in Residential Areas (in Russian). http://ohranatruda.ru/ot_biblio/normativ/data_normativ/5/5155/index.php#i643158 (accessed 04.09.13).
	Russian Ministry of Environment and Natural Resources (1993)	Russian	Russian Ministry of Environment and Natural Resources, 1993. Table 2 and Table 3, The procedure for determining the extent of damage from land contamination by chemicals (in Russian). http://www.dioxin.ru/doc/N04-25.61-5678.htm (accessed 04.09.13).
(Russia) City of **Moscow**	Moscow Government (2004)	Russian	Moscow Government, 2004. Improving the Quality of Soils in the City of Moscow (in Russian). http://www.ecoekspert.ru/art/rcent/44.html (accessed 05.09.13).
(Russia) Republic of **Tatarstan**	Republic of Tatarstan Ministry of Environment and Natural Resources (2002)	Russian	Republic of Tatarstan Minister of Ecology and Natural Resources, 2002. Order-Sizing Penalties for Any Damage to Land and Vegetation (No. 314) (in Russian). eco.tatar.ru/rus/file/pub/pub_8462.doc (accessed 05.09.13).
Republic of **Serbia**	Serbia Ministry of Environment And Spatial Planning (1994)	English	Serbia Ministry of Environment and Spatial Planning, 1994. Rulebook on the Allowed Quantities of Hazardous and Harmful Substances in the Ground and on the Methods for Their Examination. Official Herald of the Republic of Serbia, No. 23/94. http://www-wds.worldbank.org/external/default/WDSContentServer/WDSP/IB/2005/04/21/000011823_20050421141937/Rendered/INDEX/E11400VOL.06.txt (accessed 08.08.11).
Republic of **Singapore**	Singapore National Environmental Agency (2010)	English	Singapore National Environmental Agency, 2010. Guideline on Environmental Baseline Study. http://www.jtc.gov.sg/Documents/EBSGuidelines.pdf (accessed 03.09.13).
Slovak Republic (**Slovakia**)	Slovakia Ministry of Agriculture (2004)	Slovak	Slovakia Ministry of Agriculture, 2004. Act No. 220/2004 Coll. On the Protection and Use of the Agricultural Soil (in Slovak). http://www.podnemapy.sk/portal/verejnost/konsolidacia/z_220_2004.pdf (accessed 03.09.13).
Republic of **Slovenia**	Slovenia Ministry of Environment and Spatial Planning (1996)	Slovenian	Slovenia Ministry of Environment and Spatial Planning, 1996. The Limit, Warning and Critical Levels of Hazardous Substances in Soil (in Slovenian) Official Gazette of the Republic of Slovenia 68/1996, 5769-5843 (Nov. 29, 1996). http://www.uradni-list.si/_pdf/1996/Ur/u1996068.pdf (accessed 03.09.13).
Republic of **South Africa**	South Africa Department of Environmental Affairs (2010)	English	South Africa Department of Environmental Affairs, 2010. Government Gazette, 23 August 2013, National Norms and Standards for the Assessment of Waste for Landfill Disposal. https://www.environment.gov.za/sites/default/files/legislations/nemwa59of2008_norms_standards_forassessment.pdf (accessed 03.09.13).
Kingdom of **Spain**	Spain Ministry of the Presidency (2005)	Spanish	Spain Ministry of the Presidency, 2005. Royal Decree 9/2005, of 14 January, the ratio of activities set potentially contaminating soil and criteria and standards for the declaration of contaminated soils (in Spanish). http://www.boe.es/boe/dias/2005/01/18/pdfs/A01833-01843.pdf (accessed 04.09.13).
(Spain) Autonomous Community of **Andalusia**	Andalusia Ministry of Environment (2006)	Spanish	Andalusia Ministry of Environment, 2006. Evaluation of Quality Soil and Levels of Generic Reference (in Spanish). http://www.juntadeandalucia.es/medioambiente/web/Bloques_Tematicos/Estado_Y_Calidad_De_Los_Recursos_Naturales/Suelo/Contaminacion_pdf/Evaluacion.pdf (accessed 04.09.13).
(Spain) Autonomous Community of **Aragon**	Government of Aragon (2005)	Spanish	Government of Aragon, 2005. Implementation of real decree 9/2005 DE contaminated soil in Aragon (in Spanish). http://www.camarazaragoza.com/medioambiente/docs/bibliocamara/bibliocamara165.pdf (accessed 04.12.13).
(Spain) Principality of **Asturias**	The Government of the Principality of Asturias (2005)	Asturianu	The Government of the Principality of Asturias, 2005. A Preliminary Report on The Situation of Contaminated Soils (in Asturianu). http://www.asturia.es/porta;/site/Asturias/menuitem.46a76b28f520ecaaf18e90bbb30a0a0/?vgnextoid=90f3c159b5d4f010VgnVCM100000b0030a0aRCRD&i18n.http.lang=es (accessed 07.12.13).
(Spain) Autonomous Community **Balearic Islands**	Ministry of Agriculture, Environment and Territory of Balearic Islands (2013)	Catalan	Ministry of Agriculture, Environment and Territory of Balearic Islands, (2013). Contaminated soil (in Catalan). http://www.caib.es/govern/sac/visor_proc.do?codi=507019&lang=en (accessed 07.12.13).
(Spain) **Basque** Country	Basque Government, Department of Environment, Planning, Agriculture and Fisheries (2005)	Basque	Basque Government Department of Environment, Planning, Agriculture and Fisheries, 2005. Law 1/2005 for the Prevention and Correction of Soil Contamination (in Basque), Official Gazette of Basque Country No 32, Feb.16, 2005. http://www.euskadi.net/bopv2/datos/2005/02/0500661a.pdf (accessed 04.09.13).
(Spain) Autonomous Community of **Canary Islands**	Government of Canary Islands (2007)	Spanish	Government of Canary Islands, 2007. Annex I, Natural Sciences, Official Bulletin No. Canarias. 113, Thursday June 7, 2007 (in Spanish). http://www.gobcan.es/boc/2007/113/boc-2007-113-anexo-12892-13233.pdf (accessed 07.12.13).
(Spain) Autonomous Community of **Cantabria**	Government of Cantabria (2006)	Spanish	Government of Cantabria, 2006. Establishing the legal regime regulating soil contaminated in Cantabria and creates Soil Stock Contaminated (in Spanish). https://www.netenvira.com/webnormas/webnormas/noticias/proyecto-de-decreto-de-suelos-contaminados-en-cantabria_3466_966_4137_0_1_in.html (accessed 07.12.13).
(Spain) Autonomous Community of **Castile and Leon**	Government of Castile And Leon	Spanish	Government of Castile and Leon. Progress Report CONTAMINATED SOIL (in Spanish). https://www.tramitacastillayleon.jcyl.es/web/jcyl/AdministracionElectronica/es/Plantilla100DetalleFeed/1251181050732/Tramite/1277228074975/Tramite (accessed 07.12.13).
(Spain) Autonomous Community of **Castile La Mancha**	Jiménez Ballesta Et Al. (2010)	Spanish	R. Jiménez Ballesta1, P. Conde Bueno2, J.A. Martín Rubí2, R. García Giménez, 2010. Geochemical background levels and influence of the geological setting on pedogeochemical baseline concentrations of trace elements in selected soils of Castilla-La Mancha (Spain) http://webcache.googleusercontent.com/search?q=cache:e1fp7nsyNloJ:estudiosgeol.revistas.csic.es/index.php/estudiosgeol/article/download/800/828+&cd=1&hl=en&ct=clnk&gl=us (accessed 07.12.13).
(Spain) Autonomous Community of **Catalonia**	Waste Agency of Catalonia (2005)	Catalan	Waste Agency of Catalonia, 2005. Royal Decree 9/2005, of 14 January, on the list of potentially soil polluting activities, and the criteria and standards for the declaration of contaminated soils. http://www20.gencat.cat/portal/site/arc/menuitem.60fb2478680e61fd624a1d25b0c0e1a0/?vgnextoid=795d0431b17d6210VgnVCM1000008d0c1e0aRCRD&vgnextchannel=795d0431b17d6210VgnVCM1000008d0c1e0aRCRD&vgnextfmt=default (accessed 07.12.13).
	Andalusia Ministry of Environment (2006)	Spanish	Andalusia Ministry of Environment, 2006. Evaluation of Quality Soil and Levels of Generic Reference (in Spanish). http://www.juntadeandalucia.es/medioambiente/web/Bloques_Tematicos/Estado_Y_Calidad_De_Los_Recursos_Naturales/Suelo/Contaminacion_pdf/Evaluacion.pdf (accessed 04.09.13).
(Spain) Autonomous City of **Ceuta**	Official Portal of Ceuta (2013)	Spanish	Official Portal of Ceuta, 2013. Official Gazette, CITY Ceuta, Management And Administration: CITY PALACE - Archive Official Gazette City Ceuta Management and Administration: CITY PALACE - Archive Number 5,307 (in Spanish). http://www.ceuta.es/ceuta/documentos/finish/1489-octubre/7935-bocce-5307-25-10-2013 (accessed 05.12.13).
(Spain) Autonomous Community of **Extremadura**	Ministry of Agriculture and Rural Development (2010)	Spanish	Ministry of Agriculture and Rural Development, 2010. ORDER of August 3, 2010 approving the application of regulated legal compliance and management requirements are established and good agricultural and environmental conditions in the Community Autónoma de Extremadura (2010050248) (in Spanish). http://doe.juntaex.es/pdfs/doe/2010/1540o/10050248.pdf (accessed 07.12.13).
(Spain) Autonomous Community of **Galicia**	Ministry of Environment and Sustainable Development of Galicia (2009)	Galician	Ministry of Environment and Sustainable Development of Galicia, 2009. DECREE 59/2009, of 26 February, on the traceability of waste is regulated (in Spanish). http://www.xunta.es/dog/Publicados/2009/20090324/Anuncio10CC2_es.html (accessed 07.12.13).
	Andalusia Ministry of Environment (2006)	Spanish	Andalusia Ministry of Environment, 2006. Evaluation of Quality Soil and Levels of Generic Reference (in Spanish). http://www.juntadeandalucia.es/medioambiente/web/Bloques_Tematicos/Estado_Y_Calidad_De_Los_Recursos_Naturales/Suelo/Contaminacion_pdf/Evaluacion.pdf (accessed 04.09.13).
(Spain) Autonomous Community of **La Rioja**	Government of La Rioja (2007)	Spanish	Government of La Rioja, 2007. Generic reference levels (NGR) NGR for metals and protection of human health (in Spanish). http://www.larioja.org/npRioja/default/defaultpage.jsp?idtab=449537 (accessed 07.12.13).
(Spain) Autonomous Community of **Madrid**	Spain Ministry of the Presidency (2005)	Spanish	Ministry of Environment and Planning, 2007. ORDER 761/2007 of 2 April, the Minister of the Environment and Planning, by amending Order 2770/2006 of 11 August, by which generic reference levels of heavy metals and other trace elements in contaminated soils down Madrid (in Spanish). http://www.madrid.org/cs/Satellite?blobcol=urldata&blobheader=application%2Fpdf&blobkey=id&blobtable=MungoBlobs&blobwhere=1158627540041&ssbinary=true (accessed 07.12.13).
(Spain) Autonomous City of **Melilla**	Ministry of Environment of the Autonomous City of Melilla (Undated)	Spanish	Ministry of Environment of the Autonomous City of Melilla, undated. Contaminated Soil (in Spanish). http://www.melillamedioambiente.com//index.php?option=com_content&task=view&id=201&Itemid=32 (accessed 05.12.13).
(Spain) Region of **Murcia**	Government of Region of Murcia (2011)	Spanish	Government of Region of Murcia, 2011. 2025 - Declaration of contaminated soils (in Spanish). http://www.carm.es/web/pagina?IDCONTENIDO=2025&IDTIPO=240&NOMBRECANAL=Calidad+del+Aire+y+Protecci%F3n+de+la+Atm%F3sfera&RASTRO=c643$m3063,3283 (accessed 04.12.13).
(Spain) Autonomous Community of **Navarra**	Department of Rural Development, Environment and Local Government (Undated)	Basque	Department of Rural Development, Environment and Local Government, undated. State Legislation of Navarra. http://www.navarra.es/home_es/Gobierno+de+Navarra/Organigrama/Los+departamentos/Desarrollo+Rural+Industria+Empleo+y+Medio+Ambiente/Acciones/Planes+especificos/Acciones+medio+ambiente/Informacion+ambiental/Elementos/Suelos/Legislacion/Estatal.htm (accessed 13.12.13).
(Spain) Autonomous Community of **Valencia**	Generalistat Valencian Regional Ministry of Infrastructure, Planning and the Environment (2007)	Catalan	Generalistat Valencian Regional Ministry of Infrastructure, Planning and the Environment, 2007. Floor Guide Contaminated (in Spanish). http://www.cma.gva.es/contenidoHtmlArea/contenido/52721/cas/Guia_suelos.pdf (accessed 07.12.13).
United Republic of **Tanzania**	Tanzanian Bureau of Standards (2007)	English	Tanzania Bureau of Standards, 2007. Soil Quality e Limits for Soil Contaminants in Habitat and Agriculture (TSZ 972). http://www.tbs.go.tz/standards/index.php/tbs/tzs_972/ (accessed 04.04.12).
Kingdom of **Thailand**	Thailand Ministry of Natural Resources And Environment (2004)	English	Thailand Ministry of Natural Resources and Environment, 2004. Soil Quality Standards for Habitat and Agriculture. http://www.pcd.go.th/info_serv/en_reg_std_soil01.html#s1 (accessed 07.09.13).
Republic of **Turkey**	Turkey Ministry of Environment And Forestry (2001)	Turkish	Turkey Ministry of Environment and Forestry, 2001. Soil Pollution Control Regulation (in Turkish), Official Gazette of Republic of Turkey No. 24609 (10/12/2001). http://www.bcm.org.tr/pdf/toprak_kir_yon.doc (accessed 07.09.13).
**Ukraine**	Ministry of Health of Ukraine (2001)	Ukrainian	Ministry of Health of Ukraine, 2001. Appendix 2 and 3, State sanitary rules and norms DSanPiN 8.8.1.2.3.4-000-2001 (in Ukrainian). http://www.uazakon.com/documents/date_8m/pg_grwssr/index.htm (accessed 07.09.13).
(**United Kingdom**) Anglian Water Services	Anglian Water Services Ltd. (2010)	English	Anglian Water Services Ltd, 2010. Information for Developers about Contaminated Land and Ground Condition Assessment. http://www.anglianwater.co.uk/_assets/media/Contaminated-sites.pdf (accessed 07.09.13).
(United Kingdom) **White Young Green Environmental Ltd**	White Young Green Environmental Ltd. (2008)	English	White Young Green Environmental Ltd, 2008. Sainsburys Supermarkets Ltd Waterlooville Ground Condition Assessment Report, Appendix DeWYGEs TVSs. http://www5.havant.gov.uk/AcolaidIntegration/GetDocumentContent.aspx?documentId=195262 (accessed 07.09.13).
**United States**	U.S. Environmental Protection Agency (2013)	English	U.S. Environmental Protection Agency, 2013. Risk-Based Screening Table. http://www.epa.gov/reg3hwmd/risk/human/rb-concentration_table/Generic_Tables/index.htm (accessed 10.01.14).
	National Oceanic and Atmospheric Administration Office of Response and Restoration (2008)	English	National Oceanic and Atmospheric Administration Office of Response and Restoration, 2008. Screening Quick Reference Tables. http://www.gesamp.org/data/gesamp/files/file_element/4a2a322c8acb2c26cc0234685eac71fa/SQuiRTs.pdf (accessed 23.01.14).
	Boeing Company, National Aeronautics and Space Administration and Department of Energy (2010)	English	Boeing Company, National Aeronautics and Space Administration and Department of Energy, 2010. Expedited List of Rural Residential Risk-Based Screening Levels – Tables. http://www.dtsc-ssfl.com/default.asp?V_DOC_ID=910&subID=8&subID2=8 (accessed 22.01.14).
	Boeing Company, National Aeronautics and Space Administration and Department of Energy (2010)	English	Boeing Company, National Aeronautics and Space Administration and Department of Energy, 2010. Expedited List of Rural Residential Risk-Based Screening Levels – Tables. http://www.dtsc-ssfl.com/default.asp?V_DOC_ID=910&subID=8&subID2=8 (accessed 22.01.14).
	U.S. Army Center for Health Promotion and Preventive Medicine (2013)	English	U.S. Army Center for Health Promotion and Preventive Medicine, 2013. Technical Guide 230 Environmental Health Risk Assessment and Chemical Exposure Guidelines for Deployed Military Personnel. http://phc.amedd.army.mil/PHC%20Resource%20Library/TG230.pdf (accessed 23.01.14).
	Agency of Toxic Substance and Disease Registry (2008, 2009a,f, 2010c, 2013)	English	Agency of Toxic Substance and Disease Registry, 2008. Public Health Assessment for KEN FOSTER FARM (FORMER) SHERWOOD, OREGON. http://www.atsdr.cdc.gov/hac/pha/KenFosterFarm/KenFosterFarmFinalPHA040308.pdf (accessed 29.01.14).
		English	Agency of Toxic Substance and Disease Registry, 2009a. Public Health Assessment PFOHL Brothers Landfill Cheektowaga, Erie County, New York. http://www.atsdr.cdc.gov/HAC/pha/PHA.asp?docid=253&pg=0 (accessed 29.01.14).
		English	Agency of Toxic Substance and Disease Registry, 2010c. Pesticide Misapplication in a Private Residence Parsons, Labette County, KS 67357. http://www.atsdr.cdc.gov/HAC/pha/ParsonsMalathionStrikeTeam/ParsonsMalathionStrikeTeamHC1117.pdf (accessed 29.01.14).
		English	Agency of Toxic Substance and Disease Registry, 2013. Wright Chemical Corporation NPL Site Adjacent to Livingston Creek Riegelwood, Columbus County, North Carolina. http://epi.publichealth.nc.gov/oee/hace/docs/WrightChemical_ColumbusCo.pdf (accessed 29.01.14).
Republic of **Uzbekistan**	Head of State Health Officer of The Republic of Uzbekistan (2005)	Russian	Head of State health officer of the Republic of Uzbekistan, 2005. Sanitary Maximum Permissible Concentration (MPC) and the Indicative Permissible Concentration (APC) of Exogenous Harmful Substances in Soil 2005 (in Russian). http://www.lex.uz/Pages/GetAct.aspx?lact_id=1905543&search_text= (accessed 10.10.13).
Socialist Republic of **Vietnam**	Republic of Vietnam (2008)	Vietnamese	Republic of Vietnam, 2008. National technical regulation on the pesticide residues in the soils (in Vietnamese). http://vea.gov.vn/vn/vanbanphapquy/tcmt/Documents/Quy%20chuan%20ky%20thuat%20quoc%20gia%20ve%20MT/QC_HCBVTV%2004doc.pdf (accessed 08.09.13).
	Republic of Vietnam (1995)	Vietnamese	Republic of Vietnam, 1995. TCVN5941-1995 (in Vietnamese). http://thuvienkhcn.vinhlong.gov.vn/tailieukhcn/data/TieuChuantoanvan/MOITRUONG/TCVN_5941_95.pdf (accessed 22.11.13).
*Jurisdictions other than United Nations member states*			
**Union of Soviet Socialist Republics** (USSR)	The State Standard of the Ussr (1983)	Russian	The State Standard of the USSR, 1983. Soil: Classification of Chemicals for Pollution Control. 17.4.1.02-83 Standard (in Russian). http://zakonbase.ru/content/base/66884 (accessed 04.09.13).
	State Medical Officer of the USSR (1982)	Russian	State Medical Officer of the USSR, 1982. Guidelines in hygienic substantiation MPC Chemical Substances in the soil. August 5, 1982 N 2609-82 (in Russian). http://www.libussr.ru/doc_ussr/usr_11234.htm (accessed 04.09.13).
	The USSR Ministry (1991)	Russian	The USSR Ministry, 1991. The list of maximum permissible concentration (MPC) of chemicals in the soil № 6229-91. Approved. USSR Ministry of 19/11/91 (in Russian). http://pravo.levonevsky.org/baza/soviet/sssr0012.htm (accessed 04.09.13).
	Ministry Of Health of the USSR (1980)	Russian	Ministry of Health of the USSR, 1980. Maximum allowable concentrations of chemicals in the soil (MPC) (in Russian). http://www.libussr.ru/doc_ussr/usr_10508.htm (accessed 04.09.13).

**Table S2:** Worldwide pesticide drinking water regulatory jurisdictions references and dates accessed

Jurisdictions	Sources	Languages	Websites References
*Multinational organizations*			
**European Union**	European Union (1998)	English	European Union, 1998. On the quality of water intended for human consumption. http://eur-lex.europa.eu/legal-content/EN/TXT/PDF/?uri=CELEX:31998L0083&from=EN (Accessed August 18. 2014).
**World Health Organization**	World Health Organization (2011)	English	World Health Organization, 2011. Guidelines for drinking-water quality fourth edition. http://www.who.int/water_sanitation_health/publications/2011/dwq_chapters/en/ (Accessed April 5. 2014).
*United Nations member states*			
Republic of **Albania**	Albania Institute for European Environmental Policy (2007)	Albanian	Albania Institute for European Environmental Policy, 2007. Drinking water quality standards (STASH 3904:1997 ICS 13060) (In Albanian). http://ec.europa.eu/environment/archives/international_issues/pdf/report_albania.pdf (Accessed September 10. 2014).
Principality of **Andorra**	Andorra Official Gazette (1999)	Catalan	Andorra Official Gazette, 1999. Regulation for technical healthcare delivery and quality control of drinking water for human (in Catalan).https://www.bopa.ad/bopa/011044/Pagines/19D62.aspx (Accessed August. 14. 2014).
**Antigua** and **Barbuda**	Environmental Solutions Antigua Limited (2008)	English	Environmental Solutions Antigua Limited, 2008. Water Quality Guidelines for Antigua & Barbuda. http://gefantigua.org/wp-content/uploads/2010/11/Water-Quality-Guidelines-for-AB-Final_0.pdf (Accessed September 18. 2014).
**Argentine** Republic	Argentine Official Gazette (1993)	Spanish	Argentine Official Gazette 1993. Regulatory Decree Law 24,051 on hazardous waste regime (In Spanish). http://www.ambiente.gov.ar/?aplicacion=normativa&IdNorma=538&IdSeccion=22 (Accessed August. 14. 2014).
Commonwealth of **Australia**	National Health and Medical Research Council (2013)	English	National Health and Medical Research Council, 2013. Australian Drinking Water Guidelines. http://www.nhmrc.gov.au/_files_nhmrc/publications/attachments/eh52_aust_drinking_water_guidelines_update_131216.pdf (Accessed August 15. 2014).
(Australia) **Australian Capital Territory**	Australian Capital Territory Ministry of Health (2007)	English	Australian Capital Territory Ministry of Health. Health (Drinking Water) Code of Practice. http://www.legislation.act.gov.au/di/2007-62/current/pdf/2007-62.pdf (Accessed August 15. 2014).
(Australia) State of **Tasmania**	Department of Health and Human Services (1997)	English	Department of Health and Human Services, 1997.Public Health Act 1997 Drinking Water Quality Guidelines. http://www.dhhs.tas.gov.au/__data/assets/pdf_file/0013/53320/drinking_water_quality_guidelines17_nov_05.pdf (Accessed August 15. 2014).
(Australia) State of **New South Wales**	Department of Health (2014)	English	Department of Health, 2014. Drinking Water Quality. http://www.health.nsw.gov.au/environment/water/Pages/drinking-water.aspx (Accessed August 15. 2014).
(Australia) State of **Northern Australia**	Department of Health (2014)	English	Department of Health, 2014. Water Quality. http://www.health.nt.gov.au/environmental_health/water_quality/index.aspx (Accessed August 15. 2014).
(Australia) State of **Queensland**	Government of Queensland (2014)	English	Government of Queensland, 2014. Public Health Regulation. https://www.legislation.qld.gov.au/LEGISLTN/CURRENT/P/PubHealR05.pdf (Accessed August 15. 2014).
(Australia) State of **South Australia**	Government of South Australia (2011)	English	Government of South Australia, 2011. Safe Drinking Water Act. http://www.sahealth.sa.gov.au/wps/wcm/connect/Public+Content/SA+Health+Internet/Protecting+public+health/Water+quality/ (Accessed August 15. 2014).
(Australia) State of **Western Australia**	Department of Health (2014)	English	Department of Health, 2014. Australian drinking water guidelines. http://www.public.health.wa.gov.au/3/657/2/australian_drinking_water_guidelines.pm (Accessed August 15. 2014).
Republic of **Austria**	Austria Department of Health (2013)	German	Austria Department of Health, 2013. Total legislation for drinking water regulations, as amended on 22.02.2013 (In German). https://www.verbrauchergesundheit.gv.at/lebensmittel/rechtsvorschriften/oesterreich/mwasser.html (Accessed August 16. 2014).
Commonwealth of the **Bahamas**	The Bahamas Water and Sewerage Corporation (1999)	English	The Bahamas Water and Sewerage Corporation, 1999. Customer Service Guide. http://www.wsc.com.bs/WSC_Customer%20Service%20Guide_Nov2012.pdf (Accessed August 16. 2014).
People's Republic of **Bangladesh**	Amio Water Treatment Limited (2010)	English	Amio Water Treatment Limited, 2010. Bangladesh drinking water quality standards. http://www.amiowater.com/index.php?menu_id=53&exmenu=53&page=1 (Accessed September 10. 2014).
Republic of **Belarus**	Belarus Ministry of Health (2013)	Russian	Belarus Ministry of Health, 2013. On approval of the Sanitary rules and regulations “Hygienic requirements for drinking water, packaged in containers” (In Russian). http://pravo.newsby.org/belarus/postanov12/pst861.htm (Accessed August 16. 2014).
Kingdom of **Belgium**	Brussels-Capital Region Government (2014)	English	Brussels-Capital Region Government, 2014. Annual water consumption in the Brussels-Capital Region is around 60 million cubic meters. http://www.brussels.irisnet.be/living-in-brussels/environment/water-quality (Accessed August 18. 2014).
**Belize**	Belize Agricultural Health Authority (2003)	English	Belize Agricultural Health Authority, 2003. Belize Agricultural Health Authority Act. http://www.vertic.org/media/National%20Legislation/Belize/BZ_Agricultural_Health_Authority_Act.pdf (Accessed September 18. 2014).
Kingdom of **Bhutan**	Codex Alimentarius (2001)	English	Codex Alimentarius, 2001. General Standard for Bottled/Packaged Drinking Waters. www.codexalimentarius.org/input/download/standards/.../CXP_048e.pdf (Accessed September 17. 2014).
Plurinational State of **Bolivia**	Bolivia Ministry of Public Works and Services Vice of Basic Services (2004)	Spanish	Bolivia Ministry of Public Works and Services Vice of Basic Services, 2004. Potable Water – Requirements (In Spanish). http://paap.mmaya.gob.bo/__ucp/agua_saneamiento/NORMAS/NB%20512,495-496%20CalidadAgua%20-%20oct2004/NB512%20AP_Requisitos.pdf (Accessed August 18. 2014).
Republic of **Botswana**	Water Utilities Corporation (2000)	English	Water Utilities Corporation, 2000. Botswana Standard for Drinking Water Quality Specification (BOS 32:2000). http://www.limpoporak.com/_system/DMSStorage/3461en/Botswana_WQGuidelines__BOS_32_2000.pdf (Accessed September 10. 2014).
Federative Republic of **Brazil**	Brazil Ministry of Health (2004)	Portuguese	Brazil Ministry of Health, 2004. Ordinance MS No 518/2004 (In Portuguese). http://www.tratamentodeagua.com.br/a1/artigo/portaria_quinhentos_e_dezoito.pdf (Accessed August 19. 2014).
(Brazil)State of **San Paolo**	Government of State of San Paolo (2008)	Portuguese	Government of State of San Paolo, 2008. Guiding Values of Intervention State of São Paulo (in Portuguese). http://www.mma.gov.br/port/conama/processos/FE4582B1/GTAreasContApresCETESB.pdf (Accessed August 20. 2014).
Republic of **Bulgaria**	Bulgaria Ministry of Health (2001)	Bulgarian	Bulgaria Ministry of Health, 2001. Ordinance № 9 dated 16.03.2001 on the quality of water for drinking and domestic purposes (in Bulgarian). http://www.vikmontana.com/docs/NAREDBA_9_ot_16032001.pdf (Accessed August 20. 2014).
Kingdom of **Cambodia**	Cambodia Ministry of Industry Mines and Energy (2004)	English	Cambodia Ministry of Industry Mines and Energy, 2004. Drinking Water Quality Standards. http://www.rdic.org/MIME-Drinking-Water-Quality-Standards-2004-en.pdf (Accessed September 10. 2014).
**Canada**	Health Canada (2012)	English	Health Canada, 2012. Guidelines for Canadian Drinking Water Quality - Summary Table. http://www.hc-sc.gc.ca/ewh-semt/pubs/water-eau/2012-sum_guide-res_recom/index-eng.php (Accessed August 20. 2014).
(Canada) Province of **Alberta**	Alberta Health Services (2013)	English	Alberta Health Services, 2013. Water Quality Regulations (Alberta). http://www.albertahealthservices.ca/Advisories/ne-pha-water-quality-backgrounder.pdf (Accessed August 20. 2014).
(Canada) Province of **British Columbia**	British Columbia Ministry of Health (undated)	English	British Columbia Ministry of Health. Drinking Water Treatment Objectives (Microbiological) for Surface Water Supplies in British Columbia. http://www.health.gov.bc.ca/protect/dw_treatment-objectives.html (Accessed August 21. 2014).
(Canada) Province of **Newfoundland and Labrador**	Government of Newfoundland and Labrador (2013)	English	Government of Newfoundland and Labrador, 2013. Drinking Water Safety in Newfoundland and Labrador. http://www.env.gov.nl.ca/env/waterres/reports/drinking_water/annual_report_2013.pdf (Accessed August 20. 2014).
(Canada) **Northwest Territories**	Canada Northwest Territories Municipal and Community Affairs (undated)	English	Canada Northwest Territories Municipal and Community Affairs. Northwest Territories Drinking Water Quality Database. http://www.maca.gov.nt.ca/operations/water/homepage.asp (Accessed August 20. 2014).
Canada **Nova Scotia**	Government of Nova Scotia (2012)	English	Government of Nova Scotia, 2012. Nova Scotia Treatment Standards for Municipal Drinking Water Systems. http://www.novascotia.ca/nse/water/docs/Treatment_Standards_for_Municipal_Drinking_Water_Systems.pdf (Accessed August 21. 2014).
	Nova Scotia Environment and Labor (undated)	English	Nova Scotia Environment and Labor. A Guide for Municipal Water Works on How the Guidelines for Canadian Drinking Water Quality are Implemented in Nova Scotia . http://www.novascotia.ca/nse/water/docs/GuideonGuidelinesforCanadianDrinkingWaterQuality.pdf (Accessed August 21. 2014).
(Canada) **Nunavut**	National Collaborating Centre for Environmental Health (2014)	English	National Collaborating Centre for Environmental Health, 2014. Small Drinking Water Systems: Who Does What in Nunavut. http://ncceh.ca/sites/default/files/SDWS_Who_What_Nunavut.pdf (Accessed August 21. 2014).
(Canada) Province of **Ontario**	Canadian Institute for Environmental Law and Policy (2003)	English	Canadian Institute for Environmental Law and Policy, 2003. Drinking Water Quality Standards in Ontario – Are They Tough. http://cielap.org/pdf/drinkingwaterstandards.pdf (Accessed August 21. 2014).
(Canada) Province of **Prince Edward Island**	Prince Edward Island Department of Environment, Labor and Justice (2012)	English	Prince Edward Island Department of Environment, Labor and Justice, 2012. Private Water Supplies (Wells). http://www.gov.pe.ca/environment/index.php3?number=1015748&lang=E (Accessed August 21. 2014).
(Canada) Province of **Quebec**	Government of Quebec (2014)	English	Government of Quebec, 2014. Regulation respecting the quality of drinking water. http://www2.publicationsduquebec.gouv.qc.ca/dynamicSearch/telecharge.php?type=2&file=//Q_2/Q2R40_A.htm (Accessed August 21. 2014).
(Canada) Province of **Saskatchewan**	Saskatchewan Environment (2006)	English	Saskatchewan Environment, 2006. Saskatchewan's Drinking Water Quality Standards and Objectives (summarized). http://www.saskh20.ca/DWBinder/EPB207Drinking_Water_Standards_post.pdf (Accessed August 21. 2014).
(Canada) **Yukon**	Government of Yukon (2007)	English and French	Government of Yukon, 2007. Public Health and Safety Act (In English and French). http://www.gov.yk.ca/legislation/regs/oic2007_139.pdf (Accessed August 21. 2014).
Republic of **Chile**	Chile Ministry of Public Works (2005)	Spanish	Chile Ministry of Public Works, 2005. NCH 409/1 Drinking Water Quality Standard (In Spanish). http://ciperchile.cl/pdfs/11-2013/norovirus/NCh409.pdf (Accessed August 22. 2014).
People's Republic of **China**	China Department of Health (2007)	Chinese	China Department of Health, 2007. GB 5749-85 Standards for Drinking Water Quality 2007 (In Chinese). http://www.sx-watersupply.gov.cn/NewsDetails.asp?d=1557 (Accessed August 22. 2014).
Republic of **Colombia**	Colombian Institute for Technical Standards and Certification (1994)	Spanish	Colombian Institute for Technical Standards and Certification, 1994. Official standards for water quality Colombia (In Spanish) https://www.imta.gob.mx/cotennser/images/docs/NOI/Normas_oficiales_para_la_calidad_del_agua_colombia.pdf (Accessed August 22. 2014).
Republic of **Costa Rica**	Costa Rica Minister of Finance (2005)	Spanish	Costa Rica Minister of Finance, 2005. Drinking water regulation (in Spanish). http://www.aguanalisis.com/downloads/Reglamento-de-Calidad-del-Agua-Potable-32327-S-La-Gaceta-84.pdf (Accessed August 23. 2014).
Republic of **Croatia**	Croatia Ministry of Health and Social Welfare (2007)	Croatian	Croatia Ministry of Health and Social Welfare, 2007. Ordinance Health Drinking Water (in Croatian). http://narodne-novine.nn.hr/clanci/sluzbeni/339082.html (Accessed August 19. 2014).
Republic of **Cuba**	Cuba Government (1997)	Spanish	Cuba Government, 1997. Communal Hygiene Drinking Water Health Requirements (In Spanish). https://www.imta.gob.mx/cotennser/images/docs/NOI/Normas_oficiales_para_la_calidad_del_agua_cuba.pdf (Accessed September 10. 2014).
Republic of **Cyprus**	Cyprus Ministry of Agriculture, Natural Resources and Environment (1999)	English	Cyprus Ministry of Agriculture, Natural Resources, and Environment, 1999. Water Treatment And Desalination Plants. http://www.moa.gov.cy/moa/wdd/wdd.nsf/booklets_en/DAEF71F997D25DF9C2256E85004B1583/$file/pages%201-23%20(2.41MB).pdf (Accessed September 15. 2014).
**Czech** Republic	European Commission (1998)	Czech	European Commission, 1998. The quality of water intended for human consumption (In Czech). http://www.sgpstandard.cz/editor/files/on_line/styk/demo/voda/eu/1998_83_es.pdf (Accessed August 25. 2014).
Kingdom of **Denmark**	Nature Agency of Denmark (2014)	Danish	Nature Agency of Denmark, 2014. Ordinance 292 of 26/03/2014 (In Danish). http://naturstyrelsen.dk/vandmiljoe/vand-i-hverdagen/drikkevand/ (Accessed August 25. 2014).
**Dominican** Republic	Dominican Ministry of Public Health and Social Assistance (2005)	Spanish	Dominican Ministry of Public Health and Social Assistance, 2005. DECREE 42-05 Make the Rules of Drinking Water (In Spanish). http://salud.gob.do/download/docs/Decretos/Decreto42-05.pdf (Accessed September 10. 2014).
Republic of **Ecuador**	Ecuadorian Institute of Standards (2011)	Spanish	Ecuadorian Institute of Standards, 2011. Technical Standard Ecuador NTE INEN 1108: 2011 fourth review (In Spanish).https://law.resource.org/pub/ec/ibr/ec.nte.1108.2011.pdf (Accessed August 25. 2014).
Arab Republic of **Egypt**	WHO-Regional Office for the Eastern Mediterranean (2006)	English	WHO-Regional Office for the Eastern Mediterranean, 2006. A compendium of drinking-water quality standards in the Eastern Mediterranean Region. http://applications.emro.who.int/dsaf/dsa1183.pdf (Accessed September 14. 2014).
Republic of **Estonia**	Estonia Minister of Social Affairs (2013)	Estonian	Estonia Minister of Social Affairs, 2013. Drinking water quality and control requirements and analysis methods 2013 (In Estonian). https://www.riigiteataja.ee/akt/111012013002 (Accessed August 26. 2014).
Federal Democratic Republic of **Ethiopia**	World Health Organization (2010)	English	World Health Organization, 2010. Rapid Assessment of Drinking-Water Quality in the Federal Democratic Republic of Ethiopia. http://www.wssinfo.org/fileadmin/user_upload/resources/RADWQ_Ethiopia.pdf (Accessed September 16. 2014).
Republic of **Fiji**	Secretariat of the Pacific Community (2005)	English	Secretariat of the Pacific Community, 2005. Drinking Water Quality in the Pacific Island Countries: Situation Analysis and Needs Assessment. http://ict.sopac.org/VirLib/JC0181.pdf (Accessed September 22. 2014).
Republic of **Finland**	Finland Minister of Social Affairs and Health (2001)	Finish	Finland Minister of Social Affairs and Health, 2001. Social Affairs and Health Decree of the Ministry of drinking water quality standards, and surveillance studies 461/2000 (In Finish). http://www.finlex.fi/fi/laki/alkup/2001/20010401 (Accessed August 26. 2014).
**French** Republic	France Ministry of Ecology, Sustainable Development and Energy (1998)	French	France Ministry of Ecology, Sustainable Development, and Energy, 1998. DIRECTIVE 98/83/EC 1998 (In French). http://eur-lex.europa.eu/LexUriServ/LexUriServ.do?uri=OJ:L:1998:330:0032:0054:FR:PDF (Accessed August 26. 2014).
Republic of the **Gambia**	Gambia Environmental Quality Standards Board (1999)	English	Gambia Environmental Quality Standards Board, 1999. Environmental Quality Standards Regulations. http://faolex.fao.org/docs/pdf/gam95812.pdf (Accessed September 16. 2014).
**Georgia**	Georgia Ministry of Justice (2007)	Georgian	Georgia Ministry of Justice, 2007. Technical regulations for drinking water (In Georgian). http://www.momxmarebeli.ge/images/file_955911.pdf (Accessed August 26. 2014).
Federal Republic of **Germany**	Germany Federal Ministry of Justice and Consumer Protection (2001)	German	Germany Federal Ministry of Justice and Consumer Protection, 2001. Regulation on the quality of water intended for human consumption 2001 (In German). http://www.gesetze-im-internet.de/trinkwv_2001/index.html#BJNR095910001BJNE000603377 (Accessed August 26. 2014).
**Hellenic** Republic	Greece CENTRAL PUBLIC HEALTH LABORATORY (1998)	Greek	Greece Central Public Health Laboratory, 1998. Directive 98/83 RC Council Of Europe Of 3-11-1998 “On the Water Quality of Human Consumption” (In Greek). http://www.edye.gr/htm2/EFHMER_NEA_ODHGIA.htm (Accessed August 26. 2014).
Republic of **Guatemala**	Guatemala Government (1999)	Spanish	Guatemala Government, 1999. Potable Water Standard (In Spanish). http://www.ecosistemas.com.gt/docs/01-COGUANOR-NGO-29001-1a-Revision-Agua-potable.pdf (Accessed September 19. 2014).
Republic of **Honduras**	Honduras Department of health (1995)	Spanish	Honduras Department of health, 1995. Technical standard for drinking water quality (In Spanish). http://www.salud.gob.hn/transparencia/archivos/regulacion/leyes/NORMA%20TECNICA%20CALIDAD%20AGUA%20POTABLE%20%20Honduras.pdf (Accessed September 19. 2014).
Republic of **Hungary**	Hungary National Public Health and Medical Officer Service (2001)	Hungarian	Hungary National Public Health and Medical Officer Service, 2001. Regulation (October 25th) on. Drinking water quality requirements and control arrangements (In Hungarian). http://net.jogtar.hu/jr/gen/hjegy_doc.cgi?docid=A0100201.KOR (Accessed August 26. 2014).
Republic of **Indonesia**	Indonesia Government (1990)	Indonesian	Indonesia Government, 1990. Water Quality Standards in Public Waters (In Indonesian). http://muhalikhsan.blogspot.com/2013/09/standar-kualitas-air-minum-negara.html (Accessed September 18. 2014).
Republic of **Iraq**	Iraq Central Agency for Meteorology and Quality Control (2001)	Arabic and English	Iraq Central Agency for Meteorology and Quality Control, 2001. Iraqi Legislation No. (417)-2001 Standard Specification for Drinking water (In Arabic and English). http://www.oil.gov.iq/Envirnment/PDF%20files/18.pdf (Accessed September 14. 2014).
**Ireland**	Ireland EPA (2007)	English	Ireland EPA, 2007. Standards for drinking water quality. https://www.epa.ie/pubs/advice/drinkingwater/privatewatersupplieshandbook/Section%202.pdf (Accessed August 27. 2014).
State of **Israel**	Israel Ministry of Health (2000)	Hebrew	Israel Ministry of Health, 2000. Public Health Regulations (Sanitary Quality of Drinking Water) (In Hebrew). www.old.health.gov.il/.../a205_israeli_drinking_water_regulations.doc (Accessed August 27. 2014).
Republic of **Italy**	Navy Medicine (2012)	English	Navy Medicine, 2012. Environmental Final Governing Standards Italy. http://www.med.navy.mil/sites/nmcphc/Documents/environmental-programs/bumed-eqp/overseas-environmental-compliance/FGS0-Italy-2012.pdf (Accessed August 27. 2014).
	Italy Ministry of Health (2001)	Italian	Italy Ministry of Health, 2001. General Directorate of Prevention (In Italian). http://www.arpa.veneto.it/temi-ambientali/acqua/file-e-allegati/normativa-2010/Decreto_legislativo_31_2001.pdf (Accessed August 27. 2014).
**Japan**	Japan Ministry of Health, Labor and Welfare (2001)	English and Japanese	Japan Ministry of Health, Labor and Welfare, 2001. General Standard for Bottled/Packaged Drinking Waters (In English and Japanese). http://www.mhlw.go.jp/stf/shingi/2r9852000000ip55-att/2r9852000000ipsg.pdf (Accessed August 27. 2014).
Hashemite Kingdom of **Jordan**	The Jordanian Institute of Standards and Metrology (2001)	English	The Jordanian Institute of Standards and Metrology, 2001. Drinking-water Standards: JS 286:2001. https://www3.opic.gov/environment/eia/ammanwater/Annex%20C21-Drinking%20Water%20Standards.pdf (Accessed September 14. 2014).
Republic of **Kazakhstan**	Kazakhstan Government (2001)	Russian	Kazakhstan Government, 2001. SanPin 2.1.4.1074-01 Drinking Water (In Russian). http://www.aquapool.kz/html/?id=116 (Accessed August 28. 2014).
Republic of **Kiribati**	Secretariat of the Pacific Community (2005)	English	Secretariat of the Pacific Community, 2005. Drinking Water Quality in the Pacific Island Countries: Situation Analysis and Needs Assessment. http://ict.sopac.org/VirLib/JC0181.pdf (Accessed September 22. 2014).
Republic of **Korea**	Korea Ministry of Environment (2011)	English	Korea Ministry of Environment, 2011. The Drinking Water Quality Standard. http://www.apec-vc.or.kr/?p_name=database&sort=program&sort2=EP5&gotopage=28&query=view&unique_num=ED2011081273 (Accessed September 4. 2014).
State of **Kuwait**	WHO-Regional Office for the Eastern Mediterranean (2006)	English	WHO-Regional Office for the Eastern Mediterranean, 2006. A compendium of drinking-water quality standards in the Eastern Mediterranean Region. http://applications.emro.who.int/dsaf/dsa1183.pdf (Accessed September 14. 2014).
Republic of **Latvia**	Latvia Ministry of Health (2004)	Latvian	Latvia Ministry of Health, 2004. Drinking water monitoring and control (In Latvian). http://www.vi.gov.lv/lv/vides-veseliba/dzeramais-udens/dzerama-udens-uzraudziba-un-kontrole (Accessed August 30. 2014).
**Lebanese** Republic	WHO-Regional Office for the Eastern Mediterranean (2006)	English	WHO-Regional Office for the Eastern Mediterranean, 2006. A compendium of drinking-water quality standards in the Eastern Mediterranean Region. http://applications.emro.who.int/dsaf/dsa1183.pdf (Accessed September 14. 2014).
Principality of **Liechtenstein**	Liechtenstein Drinking Water Inspectorate (1999)	English	Liechtenstein Drinking Water Inspectorate, 1999. Review on the Approaches to Water Quality Monitoring and Assessment in Europe. http://dwi.defra.gov.uk/research/completed-research/reports/dwi0799.pdf (Accessed September 16. 2014).
Republic of **Lithuania**	Lithuania Ministry of Health (2003)	Lithuanian	Lithuania Ministry of Health, 2003. Lithuanian Hygiene Standard HN 24: 2003 Drinking Water Safety and Quality Requirements (In Lithuanian). http://www.am.lt/VI/article.php3?article_id=8252 (Accessed August 31. 2014).
Grand Duchy of **Luxembourg**	Luxembourg Collection of Legislation (2002)	French	Luxembourg Collection of Legislation, 2002. Water Quality for Human Consumption (In French). http://www.legilux.public.lu/leg/a/archives/2002/0115/a115.pdf (Accessed August 31. 2014).
**Malaysia**	Malaysia Ministry of Health (2010)	English	Malaysia Ministry of Health, 2010. Drinking Water Quality Standard. http://kmam.moh.gov.my/public-user/drinking-water-quality-standard.html (Accessed August 31. 2014).
Republic of **Malta**	Malta government (2009)	Maltese	Malta government, 2009. Regulations 2009 Water Intended for Consumption Human (In Maltese). http://faolex.fao.org/docs/pdf/mlt84621.pdf (Accessed August 31. 2014).
Republic of **Mauritius**	Mauritius Government Gazette (1996)	English	Mauritius Government Gazette, 1996. Drinking Water Standards. http://environment.gov.mu/English/Pages/Legislations/Standards/Drinking-water.aspx (Accessed September 16. 2014).
United **Mexican** States	Government of Mexico (1994)	Spanish	Government of Mexico, 1994. Environmental Health Water Use for Human Consumption - Permissible Limits of Quality and Treatments to be Bound Water for Drinking Water (In Spanish). https://www.gov.mt/mt/Services-And-Information/Pages/Ir-Regolamentazzjoni-Ambjentali.aspx (Accessed August 31. 2014).
**Mongolia**	Government of Mongolia (2005)	Mongolian	Government of Mongolia, 2005. Drinking water Health and safety requirements, quality and safety assessment (In Mongolian). http://estandard.mn/filebase/files/0900-2005.pdf (Accessed September 1. 2014).
Kingdom of **Morocco**	WHO-Regional Office for the Eastern Mediterranean (2006)	English	WHO-Regional Office for the Eastern Mediterranean, 2006. A compendium of drinking-water quality standards in the Eastern Mediterranean Region. http://applications.emro.who.int/dsaf/dsa1183.pdf (Accessed September 14. 2014).
Republic of **Nauru**	Secretariat of the Pacific Community (2005)	English	Secretariat of the Pacific Community, 2005. Drinking Water Quality in the Pacific Island Countries: Situation Analysis and Needs Assessment. http://ict.sopac.org/VirLib/JC0181.pdf (Accessed September 22. 2014).
Kingdom of the **Netherlands**	Government of Netherlands (2014)	Dutch	Government of Netherlands, 2014. Drinking Water Decision (In Dutch). http://wetten.overheid.nl/BWBR0030111/geldigheidsdatum_02-09-2014 (Accessed September 2. 2014).
**New Zealand**	New Zealand Ministry of Health (2008)	English	New Zealand Ministry of Health, 2008. Drinking-water Standards for New Zealand. http://www.moh.govt.nz/notebook/nbbooks.nsf/0/237C397A37EFADA9CC25750600720E08/$file/drinking-water-standards-2008.pdf (Accessed September 2. 2014).
Republic of **Nicaragua**	Nicaragua Ministry of Health (1994)	Spanish	Nicaragua Ministry of Health, 1994. Water Quality Standards for Consumer Human (In Spanish). https://www.imta.gob.mx/cotennser/images/docs/NOI/Normas_oficiales_para_la_calidad_del_agua_nicaragua.pdf (Accessed September 19. 2014).
Federal Republic of **Nigeria**	Standards Organization of Nigeria (2007)	English	Standards Organization of Nigeria, 2007. Nigerian Standard for Drinking Water Quality. http://www.unicef.org/nigeria/ng_publications_Nigerian_Standard_for_Drinking_Water_Quality.pdf (Accessed September 2. 2014).
Kingdom of **Norway**	Norway Ministry of Health and Care Services (2001)	Norwegian	Norway Ministry of Health and Care Services, 2001. Regulations on water supply and drinking water (Drinking Water Regulations) (In Norwegian). http://lovdata.no/dokument/SF/forskrift/2001-12-04-1372 (Accessed September 2. 2014).
Islamic Republic of **Pakistan**	Pakistan Standards and Quality Control Authority (undated)	English	Pakistan Standards and Quality Control Authority. Drinking Water Quality Guidelines and Standards. http://www.pcrwr.gov.pk/Press%20Releases%202012/COMSTECH%20Workshop/Ms%20Saiqa%20Imran/05-Water%20Quality%20Standards.pdf (Accessed September 11. 2014).
Republic of **Palau**	Environmental Quality Protection Board (undated)	English	Environmental Quality Protection Board. Public Water Supply System Regulations. http://faolex.fao.org/docs/pdf/pau32797.pdf (Accessed September 22. 2014).
Republic of **Peru**	Peru Ministry of Health (2011)	Spanish	Peru Ministry of Health, 2011. Regulation of Water Quality Human Consumption (In Spanish). http://www.digesa.minsa.gob.pe/publicaciones/descargas/reglamento_calidad_agua.pdf (Accessed September 3. 2014).
Republic of the **Philippines**	Philippines Department of Health (2007)	English	Philippines Department of Health, 2007. Philippine National Standards for Drinking Water. http://www.lwua.gov.ph/downloads_14/Philippine%20National%20Standards%20for%20Drinking%20Water%202007.pdf (Accessed September 18. 2014).
Republic of **Poland**	Poland Ministry of Health (2007)	Polish	Poland Ministry of Health, 2007. The quality of water intended for human use (In Polish). http://www.wodociagiostrzeszowskie.pl/asp/pliki/pobierz/rozporzadzenie-_jakosc_wody.pdf (Accessed September 3. 2014).
**Portuguese** Republic	Portugal Ministry of Environment, Planning and Regional Development (2007)	Portuguese	Portugal Ministry of Environment, Planning and Regional Development, 2007. Gazette, 1st Series - No. 164 - August 27, 2007 (In Portuguese). http://dre.pt/pdf1s/2007/08/16400/0574705765.pdf (Accessed September 3. 2014).
State of **Qatar**	The Gulf Cooperation Council (GCC) Standardization (2012)	Arabic and English	The Gulf Cooperation Council (GCC) Standardization, 2012. Bottled drinking water (In Arabic and English). http://old.sfda.gov.sa/NR/rdonlyres/144C70B0-4648-4A6E-A06C-99EEB6FB8772/0/%D9%85%D9%8A%D8%A7%D9%87%D8%A7%D9%84%D8%B4%D8%B1%D8%A8%D8%A7%D9%84%D9%85%D8%B9%D8%A8%D8%A7%D8%A9%D8%AE%D9%84%D9%8A%D8%AC%D9%8A%D8%A7%D9%86%D8%AC%D9%84%D9%8A%D8%B2%D9%8A1.pdf (Accessed September 3. 2014).
**Russian** Federation	Russian Ministry of Health (2002)	Russian	Russian Ministry of Health, 2002. DRINKING WATER.HYGIENE REQUIREMENTS FOR QUALITY WATER SanPin 2.1.4.1074-01 (In Russian). http://ross-water.com/files/sanpin/file-2.pdf?1242648352 (Accessed August 28. 2014).
	Russian Ministry of Health (2002)	Russian	Russian Ministry of Health, 2002. Drinking water. Hygienic requirements for water quality, packaged in a container Quality control. SanPin 2.1.4.1116-02 (In Russian). http://www.lin.irk.ru/new/files/2141116-02.pdf (Accessed August 28. 2014).
	Russian Ministry of Health (2007)	Russian	Russian Ministry of Health, 2007. Maximum permissible concentration (MPC) CHEMICALS in Water drinking and cultural and COMMUNITY WATER (In Russian). http://www.ohranatruda.ru/ot_biblio/normativ/data_normativ/41/41363/index.php (Accessed August 28. 2014).
	Russian Ministry of Health (1999)	Russian	Russian Ministry of Health, 1999. Maximum permissible concentration (MPC) of chemicals in water bodies drinking and cultural and community water Hygienic standards GN 2.1.5.2280-07 (In Russian). http://www.ohranatruda.ru/ot_biblio/normativ/data_normativ/51/51955/index.php (Accessed August 28. 2014).
	Russian Ministry of Health (1998)	Russian	Russian Ministry of Health, 1998. Drinking Water. Hygienic requirements for quality of centralized drinking water supply. Quality Control SanPin 2.1.4.559-96 (In Russian). http://www.opengost.ru/iso/13_gosty_iso/13060_gost_iso/1306020_gost_iso/3989-mu-2.1.4.682-97-metodicheskie-ukazaniya-po-vnedreniyu-i-primeneniyu-sanitarnyh-pravil-i-norm-sanpin-2.1.4.559-96-pitevaya-voda.html (Accessed August 28. 2014).
Republic of **Rwanda**	Rwanda Standards Board (2013)	English	Rwanda Standards Board, 2013. Drinking Water Standards to be Finalized In EAC Region. http://www.rwanda-standards.org/∼rbs/main-nav/media-room/news-and-events/news-details/article/drinking-water-standards-to-be-finalised-in-eac-region-supfont-colorrednewfontsup.html (Accessed September 17. 2014).
**Saint Lucia**	Caricom Regional Organization for Standards and Quality (undated)	English	Caricom Regional Organization for Standards and Quality. Guidelines for Recreational Water Quality. http://www.ssb.sr/smartcms/downloads/dcrs%2036%20guidelines%20for%20recreational%20water%20qualityDRAFT.pdf (Accessed September 21. 2014).
Republic of **Serbia**	Serbia Official Gazette (1999)	English	Serbia Official Gazette, 1999. The hygienic quality of drinking water. www.zjz.org.rs/wp-content/uploads/2013/04/pravilnik-o-higijenskoj-ispravnosti-vode-za-pice.pdf+&cd=1&hl=en&ct=clnk&gl=us (Accessed August 19. 2014).
Republic of **Singapore**	Government of Singapore (2008)	English	Government of Singapore, 2008. Environmental Public Health (Quality of Piped Drinking Water) Regulations. http://www.wsportal.org/uploads/IWA%20Toolboxes/WSP/EPH%20Regulations.pdf (Accessed September 5. 2014).
**Slovak** Republic	Council Regulation Government of the Slovak Republic (2010)	Slovak	Council Regulation Government of the Slovak Republic, 2010. Laying down requirements for water intended for human consumption and control of the quality of water intended for human consumption (In Slovak). http://www.ecoli.sk/files/documents/nv_496_2010.pdf (Accessed September 5. 2014).
Republic of **Slovenia**	Slovenia Ministry of Health (2004)	Slovenian	Slovenia Ministry of Health, 2004. RULES of drinking water (In Slovenian). http://www.uradni-list.si/files/RS_-2004-019-00865-OB∼P001-0000.PDF#!/pdf (Accessed September 5. 2014).
Republic of **South Africa**	South Africa Department of water and sanitation (2005)	English	South Africa Department of water and sanitation, 2005. Drinking Water Quality Management Guide for Water Services Authorities. http://www.dwaf.gov.za/Documents/Other/DWQM/DWQMWSAGuideSept05.pdf (Accessed September 7. 2014).
Kingdom of **Spain**	Government of Spain (2003)	Spanish	Government of Spain, 2003. Quality drinking water by the health criteria are established (In Spanish). http://www.aguaplaza.com/download/agua_potable_consumo_humano_rd_140_2003.pdf (Accessed September 7. 2014).
Republic of the **Sudan**	WHO-Regional Office for the Eastern Mediterranean (2006)	English	WHO-Regional Office for the Eastern Mediterranean, 2006. A compendium of drinking-water quality standards in the Eastern Mediterranean Region. http://applications.emro.who.int/dsaf/dsa1183.pdf (Accessed September 14. 2014).
Kingdom of **Sweden**	Sweden Nutrition and Food Agency, 2001	Swedish	Sweden Nutrition and Food Agency, 2001. NFA regulations on drinking water (In Swedish). http://www.svensktvatten.se/Documents/Kategorier/Dricksvatten/Lagar%20och%20f%C3%B6reskrifter/F%C3%B6reskrifter%20dricksvatten%202011_3.pdf (Accessed September 7. 2014).
**Swiss** Confederation	Switzerland Department of Consumer and Veterinary (2014)	French	Switzerland Department of Consumer and Veterinary, 2014. Chemical standards for drinking water Federal Ordinance on foreign substances and components (In French). http://www.vd.ch/fileadmin/user_upload/organisation/dse/scav/sire/Eaux_potables_Normes_Composition_chimique.pdf (Accessed September 7. 2014).
**Syrian** Arab Republic	WHO-Regional Office for the Eastern Mediterranean (2006)	English	WHO-Regional Office for the Eastern Mediterranean, 2006. A compendium of drinking-water quality standards in the Eastern Mediterranean Region. http://applications.emro.who.int/dsaf/dsa1183.pdf (Accessed September 14. 2014).
United Republic of **Tanzania**	Tanzania Bureau of Standards (2009)	English	Tanzania Bureau of Standards, 2009. Tanzania Water Quality Standards. http://www.frameweb.org/CommunityBrowser.aspx?id=4408 (Accessed September 7. 2014).
Kingdom of **Thailand**	Thailand Ministry of Health (2001)	Thai	Thailand Ministry of Health, 2001. (No. 256) BE 2545 Drinking Water in sealed containers (No. 4) (In Thai). http://plan.dgr.go.th/school/5.pdf (Accessed September 7. 2014).
Kingdom of **Tonga**	Secretariat of the Pacific Community (2005)	English	Secretariat of the Pacific Community, 2005. Drinking Water Quality in the Pacific Island Countries: Situation Analysis and Needs Assessment. http://ict.sopac.org/VirLib/JC0181.pdf (Accessed September 22. 2014).
Republic of **Tunisia**	Global water and wastewater quality regulations (2012)	English	Global water and wastewater quality regulations, 2012. 57.1 Regulatory framework for drinking water, wastewater discharge and reuse. http://www.globalwaterintel.com/client_media/uploaded/Global%20Regulation/Sample_Chapter.pdf (Accessed September 12. 2014).
**Tuvalu**	Secretariat of the Pacific Community (2005)	English	Secretariat of the Pacific Community, 2005. Drinking Water Quality in the Pacific Island Countries: Situation Analysis and Needs Assessment. http://ict.sopac.org/VirLib/JC0181.pdf (Accessed September 22. 2014).
Republic of **Uganda**	Uganda Ministry of Tourism, Trade and Industry (2008)	English	Uganda Ministry of Tourism, Trade and Industry, 2008. Drinking (potable) Water Specification. https://law.resource.org/pub/ug/ibr/us.201.2008.html (Accessed September 17. 2014).
**Ukraine**	Ukraine Water Health (undated)	Russian	Ukraine Water Health. Drinking water quality standards in Ukraine (In Russian). http://water-health.org.ua/?Standarty_kachestva_pitmzevoi_vody:Normativy_kachestva_pitmzevoi_vody_v_Ukraine (Accessed September 8. 2014).
**United Kingdom** of Great Britain and Northern Ireland	United Kingdom Drinking Water Inspectorate (2000)	English	United Kingdom Drinking Water Inspectorate, 2000. Guidance on the Implementation of the Water Supply (Water Quality) Regulations 2000 (as amended) IN ENGLAND. http://dwi.defra.gov.uk/stakeholders/guidance-and-codes-of-practice/WS(WQ)-regs-england2010.pdf (Accessed September 8. 2014).
**United States**	U.S. Environmental Protection Agency (2009)	English	U.S. Environmental Protection Agency, 2009. National Primary Drinking Water Regulations List of Contaminants and their (MCLs). http://water.epa.gov/drink/contaminants/index.cfm#Organic (Accessed March 22. 2014).
Eastern Republic of **Uruguay**	Uruguay Administration of Sanitary Works (2006)	Spanish	Uruguay Administration of Sanitary Works, 2006. Internal Quality Standard Drinking Water (In Spanish). http://www.ose.com.uy/descargas/reclutamiento/norma_interna_de_calidad_agua_potable_de_OSE.pdf (Accessed September 8. 2014).
Republic of **Uzbekistan**	Uzbekistan Ministry of Health (2006)	Russian	Uzbekistan Ministry of Health, 2006. Health Criteria and Control Water Quality of Centralized Drinking Water Supply of the Uzbek Population (In Russian). http://www.lex.uz/pages/GetAct.aspx?lact_id=1934624 (Accessed September 9. 2014).
Republic of **Vanuatu**	Secretariat of the Pacific Community (2005)	English	Secretariat of the Pacific Community, 2005. Drinking Water Quality in the Pacific Island Countries: Situation Analysis and Needs Assessment. http://ict.sopac.org/VirLib/JC0181.pdf (Accessed September 22. 2014).
	Secretariat of the Pacific Community (2005)	English	
Bolivarian Republic of **Venezuela**	Venezuela Ministry of Health and Welfare (1998)	Spanish	Venezuela Ministry of Health And Welfare, 1998. Health Standards for Drinking Water Quality (In Spanish). http://www.bvsde.paho.org/bvsacg/e/cd-cagua/normas/lac/20.VEN/01.norma.pdf (Accessed September 9. 2014).
Socialist Republic of **Viet Nam**	Vietnam Ministry of Health (2002)	Vietnamese	Vietnam Ministry of Health, 2002. Water hygiene standards (In Vietnamese). http://thuvienphapluat.vn/archive/Quyet-dinh-1329-2002-Q%C3%90-BYT-Tieu-chuan-ve-sinh-nuoc-an-uong-vb15749.aspx (Accessed September 9. 2014).
*Jurisdictions other than United Nations member states*			
**Palestine**	Palestinian Water Authority (1997)	Arabic	Palestinian Water Authority, 1997. Measurements of the quality of drinking water in Palestine (In Arabic). site.iugaza.edu.ps/amughari/files/Palestinian-Drinking-water-Quality-stndrds.doc+&cd=1&hl=en&ct=clnk&gl=us (Accessed September 11. 2014).
**Union of Soviet Socialist Republics** (USSR)	Medical Officer of the USSR (1981)	Russian	Medical Officer of the USSR, 1981. Guidelines for Hygiene Control Products from Synthetic Materials Offered for Use in Practice Drinking-water supply (In Russian). http://www.libussr.ru/doc_ussr/usr_10683.htm (Accessed August 29. 2014).
	State Sanitary of the USSR (1987)	Russian	State Sanitary of the USSR, 1987. Instruction Sanitary and Chemical Research Products of Plastics, Used for Use in Domestic and Drinking Water Supply and Water Resources (In Russian). http://www.libussr.ru/doc_ussr/usr_13837.htm (Accessed August 29. 2014).
